# Can ERAP1 and ERAP2 Form Functional Heterodimers? A Structural Dynamics Investigation

**DOI:** 10.3389/fimmu.2022.863529

**Published:** 2022-04-20

**Authors:** Athanasios Papakyriakou, Anastasia Mpakali, Efstratios Stratikos

**Affiliations:** ^1^ Institute of Biosciences and Applications, National Centre for Scientific Research “Demokritos”, Athens, Greece; ^2^ Laboratory of Biochemistry, Department of Chemistry, National and Kapodistrian University of Athens, Athens, Greece

**Keywords:** aminopeptidase, antigen processing, antigen presentation, enzyme mechanism, molecular dynamics, binding free energy, adaptive immunity, MHC class I

## Abstract

Endoplasmic reticulum aminopeptidases 1 and 2 (ERAP1 and ERAP2) play important roles in the generation of antigenic peptides presented by Major Histocompatibility Class I (MHCI) molecules and indirectly regulate adaptive immune responses. Although the discrete function of these enzymes has been extensively characterized, recent reports have suggested that they can also form heterodimers with functional consequences. However, lack of structural characterization of a putative ERAP1/ERAP2 dimer has limited our understanding of its biological role and significance. To address this, we employed computational molecular dynamics calculations to explore the topology of interactions between these two, based on experimentally determined homo-dimerization interfaces observed in crystal structures of ERAP2 or homologous enzymes. Our analysis of 8 possible dimerization models, suggested that the most likely ERAP1/ERAP2 heterodimerization topology involves the exon 10 loop, a non-conserved loop previously implicated in interactions between ERAP1 and the disulfide-bond shuffling chaperone ERp44. This dimerization topology allows access to the active site of both enzymes and is consistent with a previously reported construct in which ERAP1 and ERAP2 were linked by Fos/Jun zipper tags. The proposed model constitutes a tentative structural template to help understand the physiological role and significance of ERAP1/ERAP2 molecular interactions.

## Introduction

### Roles of ERAPs in Antigen Processing and Presentation

Cellular adaptive immune responses are driven by recognition of small peptides bound by Major Histocompatibility Class I molecules on the cell surface by specialized receptors on CD8^+^ T-lymphocytes. These peptides (called antigenic peptides when they elicit immune responses) are generated inside the cell by the proteolytic digestion of endogenous or internalized proteins or defective ribosomal products (1–3). While many of those proteins are initially degraded by the proteasome, the final proteolytic steps that are necessary to generate antigenic peptides are performed inside the ER, by the ER-resident specialized aminopeptidases ERAP1 and ERAP2 (4). ERAP1 and ERAP2 are ~100 kDa, highly-homologous Zn(II) aminopeptidases that can both help generate antigenic peptides from N-terminally elongated precursors and also “destroy” some antigenic peptides by over-trimming them to lengths too short to allow binding onto MHCI. By this function ERAP1 and ERAP2 can regulate the cellular immunopeptidome (the sum of peptides presented by MHC molecules) and indirectly regulate adaptive immunity, thus making them potential targets for the pharmaceutical enhancement of anti-cancer immunity or the control of inflammatory autoimmunity (5, 6).

### Function and Structure of ERAPs

The function of ERAP1 and ERAP2 has been the subject of intense research during the last 15 years and has yielded significant insight on their biological roles. The two enzymes have about 50% sequence homology and utilize a similar mechanism for trimming peptides. Several crystal structures have been solved, namely, structures in complex with substrates, substrate analogues and inhibitors (7, 8). These crystal structures have revealed a similar configuration for both enzymes that comprise of 4 structural domains arranged in a concave orientation around the active site. ERAP1 has been crystallized in two discrete conformations, in which the relative configuration of the four domains changes to either expose or shelter a large internal cavity from the external solvent (9, 10). This internal cavity can accommodate large peptide substrates and a conformational change between these two states is a key component of the catalytic cycle (11, 12). ERAP2 has only been crystallized in a “closed” conformation in which the internal cavity is not accessible to the external solvent, thus making a conformational change, like the one observed in ERAP1, obligatory for product-substrate exchange (13).

While both enzymes exhibit a similar overall structure and mechanism, they present significant differences that suggest distinct roles in antigen processing. They have distinct preferences for N-terminal amino acids (ERAP1 for hydrophobic, ERAP2 for positively charged) and substrate length (ERAP1 prefers peptides longer than 9 amino acids, while ERAP2 can efficiently trim shorter peptides) (13, 14) and have distinct effects on the cellular immunopeptidome, potentially due to differences in their internal cavity that determines enzyme–substrate interactions (15). These differences could allow them to synergize in trimming peptides in the ER so as to cover as many different sequences as possible. Indeed, synergism between ERAP1 and ERAP2 that facilitates the production of several epitopes has been demonstrated in several cases (16, 17). Finally, ERAP1 and ERAP2 are both polymorphic, with coding single nucleotide polymorphisms affecting their function and contribute to the variability of immune responses in natural populations (18, 19). Overall, ERAP1 is considered to be dominant in terms of roles in antigen processing with ERAP2 having an important but accessory or supplemental role (20). Due to their high specialization and important biological role, both enzymes are currently emerging drug-targets for cancer immunotherapy and HLA-associated inflammatory autoimmunity (6, 21, 22).

### Evidence for an ERAP1/ERAP2 Heterodimer

While most research on ERAP1 and ERAP2 has focused on their distinct roles in adaptive immunity, some studies have reported their ability to form heterodimers. An initial study detected ERAP1 and ERAP2 co-eluting after microsome fractionation, in a 230 kDa fraction as determined by density gradient centrifugation, suggesting the formation of a heterodimer (16). This finding was later re-enforced by proximity ligation assay analysis that suggested a physical interaction between the two enzymes *in situ* (23). Despite this finding however, no native recombinant stable ERAP1/ERAP2 dimer has been reported to date. To explore if this dimerization has functional consequences, researchers generated recombinant ERAP1/ERAP2 dimers stabilized by a jun/fos dimerization domain (23). Enzymatic analysis of this construct suggested a more efficient trimming function, primarily through the enhancement of ERAP1 activity (23). Using the same construct, researchers also demonstrated trimming of peptides pre-bound onto MHCI, although this mechanism was later brought into doubt (24–26). Regardless, a possible dimerization between ERAP1/ERAP2 could be the basis for enhanced synergism between the enzymes which helps define the human immunopeptidome (5).

### Crystallographic Homodimers and Scope of Study

Lack of structural information on the putative ERAP1/ERAP2 dimerization is currently limiting our ability to form a correct functional framework that includes all the components that shape the human immunopeptidome. Although no ERAP1/ERAP2 co-crystal structures have been reported, homodimerization has been observed in ERAP2 crystals and in the homologous IRAP which forms a stable dimer in solution (7, 27). Thus, we decided to utilize these experimentally determined homo-dimerization interfaces using computational molecular dynamics to explore the topology of a putative ERAP1/ERAP2 heterodimer. By analyzing 8 possible dimerization topologies, we were able to rank them in terms of predicted stability and propose the most likely topology. Our proposed model can constitute a structural template to help understand the physiological role and significance of ERAP1/ERAP2 interactions.

## Materials and Methods

### Crystallographic Structures

To investigate the structure of the putative ERAP1/ERAP2 heterodimer, we employed coordinates of the highest resolution crystal structures of ERAP1 [PDB ID: 6q4r, resolved at 1.6 Å (28)] and ERAP2 (PDB ID: 5ab0, resolved at 2.5 Å [13)]. For comparison, we also employed the X-ray crystal structures of IRAP [PDB ID: 5mj6, resolved at 2.5 Å (29)] and of human aminopeptidase N, hereafter APN [PDB ID: 4fyt resolved at 1.8 Å (30)]. Only chain A of the PDB files and side-chain atoms with the highest occupancy in case of alternate conformations have been retained. To keep the active site of the enzymes occupied and as consistent as possible, we added the ligand H-hPhe–Ψ{PO_2_CH_2_}–Leu-NH_2_, a phosphinic dipeptide analogue where hPhe is l-homo-phenylalanine and Ψ stands for the pseudopeptide bond. Coordinates of the ligand were taken from the X-ray structure of ERAP1 in complex with a 10-mer phosphinic peptide (residue KF2 in PDB ID: 6rqx) (8), including the amide nitrogen of the following residue to obtain the amidated l-Leu (hereafter, the PSE ligand). Missing atoms and residues were modeled using Modeller 9.1 (31) and protonation states of histidine residues were calculated using the H++ server (32) at physiological conditions (pH 7.4, ionic strength 0.15 M) and the default dielectric constants. For ERAP1, the latest high-resolution X-ray structures have been obtained using a construct where the exon 10 loop (residues 486−513) has been substituted by a short –GSG– linker. Considering the implication of exon 10 loop in a potential heterodimeric interface (see below), we also modeled these missing residues of ERAP1 using the structure of ERAP2 as template. At a final step, crystallographically resolved sugar moieties were added in each enzyme (**Supplementary Figure S1**).

### Preparation of the Systems

Four crystallographic dimers were used as templates to investigate putative topologies of the ERAP1/ERAP2 dimer. The first obvious choice was the crystallographic dimer of ERAP2 as resolved in the PDB ID: 5ab0 (hereafter, model A). However, we observed that except for the homodimer in the asymmetric unit, a homodimer with a distinct topology is formed between one chain of the asymmetric unit and a symmetry-related chain along the –z axis (**Supplementary Figure S3**), an interaction that could not be identified in the initial X-ray structures of ERAP2 due to weaker electron density at this interface. Thus, we also used this alternative ERAP2 homodimer as template for modeling ERAP1/ERAP2 heterodimer (hereafter, model B). It should be noted that in the crystallographic ERAP2 dimer the exon 10 loop region is visible only in one of the two molecules of the asymmetric unit, i.e., chain A of PDB ID: 5ab0. Therefore, we used the coordinates of this region from chain A to construct both ERAP2 homodimeric models A and B. Two additional templates were employed from the homologous aminopeptidases IRAP (hereafter model C) and APN (model D), both of which form homodimers. For each template, ERAP1 and ERAP2 were superimposed on each chain of the homodimer separately to generate two similar, but not identical, topologies. In this way, four sets of ERAP1/ERAP2 heterodimers (designated as models A1, B1, C1, D1) and four sets of ERAP2/ERAP1 heterodimers (designated as models A2, B2, C2, D2) were generated. In a few cases, extended clashes of the attached carbohydrates were manually fixed by rotating the glycosidic torsions appropriately.

For the simulations of the jun/fos-linked heterodimer, we employed the X-ray structure of the heterodimer of the bZIP regions of c-Fos and c-Jun bound to DNA (PDB ID: 1fos) (33), from which chains E (c-Fos) and F (c-Jun) were taken. The heterodimeric ERAP1/ERAP2 models were initially placed manually in an appropriate orientation from the jun/fos dimer, so that the C-termini residues of ERAP1 and ERAP2 are aligned with the N-termini of c-Jun and c-Fos, respectively. Then the LoopModel method of Modeller was used to optimize the linker residues between enzymes and the jun/fos helices, while keeping the heterodimeric structures intact. The sequences used to model the ERAP1–c-Jun/ERAP2–c-Fos dimers can be found in **Supplementary Table S1**.

The simulation systems were prepared using the LEaP module of AMBER v18 (34) with protein force field parameters from the ff14SB parameter set (35). Force field parameters for the phosphinic dipeptide PSE ligand (H-hPhe–Ψ{PO_2_CH_2_}–Leu-NH_2_) were generated using ANTECHAMBER with GAFF parameters (36) and AM1-BCC atomic charges (37). The catalytic zinc was bonded with the two histidine and the glutamate residues in the active site and an additional bond with the phosphinic oxygen of PSE was created. Geometry around Zn(II) was retained as tetrahedral with 4 single bonds (*r*_eq_ = 2.1 Å, *k*_r_ = 150 kcal mol^−1^ Å^−2^) and all angles at *θ*_eq_ = 109.5 deg with *k*_θ_ = 50 kcal mol^−1^ rad^−2^. Sugar moieties were modeled using the residue names shown in **Supplementary Figure S1** and parameters from the GLYCAM06j force field (38). The glycan-linked Asn residues were converted to NLN and disulfide bonds were created between adjacent cysteine (CYX) residues. All dimers were solvated in truncated octahedral TIP3P water boxes with a minimum buffer of 10 Å around the solute, and then the required number of Na^+^/Cl^−^ ions were added to ensure neutrality of the system. In total, 12 solvated systems were generated, the 4 homodimeric templates and 8 ERAP1/ERAP2 heterodimeric models, which comprised a total number of 190–280 thousands of atoms depending on the topology of the dimer. Two additional systems comprising ERAP1–c-Jun/ERAP2–c-Fos heterodimer were generated using the same procedure.

### Molecular Dynamics Simulations

Molecular dynamics (MD) simulations were carried out using the GPU-accelerated PMEMD module of AMBER v18 (39). The integration time step was 2 fs, in combination with the SHAKE algorithm to constraint hydrogen atoms at their equilibrium distance. Long-range electrostatic interactions were treated using the particle mesh Ewald summation method with a tolerance of 10^−6^ and the real space cut-off was set to 10 Å. The Langevin thermostat was used to regulate the target temperature and the Berendsen weak-coupling algorithm to regulate the pressure of the system. The relaxation time of the barostat was set 1.0 ps and the collision frequency of the thermostat was set to 1.0 ps^−1^, unless indicated. Equilibration of the systems and production simulations were performed as described in recent works (8). Briefly, after energy minimization of the systems with 1,000 steps with the steepest descent algorithm followed by 9,000 steps of the conjugate gradient method, or until the energy gradient was <0.1 kcal mol^−1^ Å^−1^, the systems were heated to 200 K *via* a short simulation of 10 ps under constant volume (*NVT* ensemble). Positional restraints with a force constant of 100 kcal mol^−1^ Å^−2^ were applied to all non-hydrogen atoms. The system was then equilibrated at the target temperature and pressure (*T* = 300 K, *P* = 1 atm) for 100 ps under constant pressure (*NPT* ensemble), while applying restraints only on C^α^ atoms of the proteins. Restraints were then gradually relaxed *via* 4 rounds of 100-ps *NPT* simulation (10 → 1.0 → 0.1 → 0.01 kcal mol^−1^ Å^−2^), followed by 9.5 ns of unrestraint *NPT* simulations (*T* = 300 K, *P* = 1 atm). Production runs were carried out under the same conditions for 100 ns, starting from the 10-ns equilibrated systems. To increase sampling of the conformational space, we performed triplicate simulations of each system by varying the friction coefficient of the thermostat (*γ* was set to 1.0, 0.5, and 0.1 ps^−1^). Trajectories were updated every 5 ps for a total of 20,000 snapshots for each production run.

### Analysis of the Trajectories

Trajectory processing and analysis has been carried out using the CPPTRAJ module of AMBER v18 (40). Root–mean–square deviation (RMSD) of C^α^ atoms were calculated with respect to the initial coordinates after RMS fitting. Clustering of the trajectories was carried out with the hierarchical agglomerative approach implemented in CPPTRAJ, using a minimum distance between clusters of 2.0 Å with respect to the C^α^ atoms of the dimers. In addition, 100 frames for each simulation were sampled uniformly (every 1 ns) from the total of 20,000 frames for statistical analysis. Solvent and counterions, sugar moieties, Zn(II) and the bound ligand were removed to retain only the protein atoms. The buried surface area (BSA) of the dimer was calculated using the MSMS module in VMD v1.9 (41) with a 1.4-Å radius sphere, by extracting the solvent-accessible surface area (SASA) of the dimer from the sum of the SASA of the two separated bound protomers. The binding affinity of the dimers was calculated by means of estimated free energy of binding using PRODIGY v2.1 (42). Briefly, the binding affinity is calculated using the following simple linear equation:


$$\Delta G_{\text{calc}}=0.09459\;\text{I}{\text{C}}_{\text{charged}/\text{charged}}+0.10007\text{I}{\text{C}}_{\text{charged}\_\text{apolar}}-0.19577\text{I}{\text{C}}_{\text{polar}/\text{polar}}+0.22671\text{I}{\text{C}}_{\text{polar}/\text{apolar}}-0.18681\%\text{NI}{\text{S}}_{\text{apolar}}-0.13810\%\text{NI}{\text{S}}_{\text{charged}}+15.9433$$

where, **IC_(aa type)_** is the number of interface residue pair-wise contacts between different types of amino acids (charged/charged, charged/apolar, polar/polar and polar/apolar), and **%NIS_(aa type)_** is the percent of apolar or charged residues on the non-interacting surface from the total number of residues on the non-interacting surface (polar residues: C, H, N, Q, S, T, Y, W; apolar residues: A, F, G, I, V, M, P; charged residues: E, D, K, R). The reported BSA (Å^2^) and Δ*G* ^est^ (kcal/mol) in **Tables 2**, **3** are mean values from 100 snapshots taken from the MD trajectories of the dimers. Figures were generated using VMD v1.9 or PyMOL v2.3 (open-source build).

**Table 1:** Summary of the systems employed in the MD calculations based on the 4 crystallographic homodimers A–D.

ID	System topology	BSA (Å^2^)	Δ*G* ^est^ (kcal/mol)	*K*_d_ (μM)	IM contacts
**A**	**ERAP2 homodimer**	**1,195.7**	**−9.8**	**0.070**	**52**
A1	ERAP1/ERAP2	1,232.1	**−**9.4	0.130	51
A2	ERAP2/ERAP1	1,260.6	**−**9.6	0.096	52
**B**	**ERAP2 homodimer**	**1,605.6**	**−11.8**	**0.002**	**62**
B1	ERAP1/ERAP2	1,975.6	**−**8.3	0.850	77
B2	ERAP2/ERAP1	1,313.0	**−**10.4	0.022	49
**C**	**IRAP homodimer**	**2,609.6**	**−11.5**	**0.004**	**84**
C1	ERAP1/ERAP2	1,988.7	**−**9.1	0.200	75
C2	ERAP2/ERAP1	2,004.0	**−**8.9	0.290	68
**D**	**APN homodimer**	**1,775.1**	**−10.6**	**0.017**	**71**
D1	ERAP1/ERAP2	2,375.1	**−**9.6	0.084	87
D2	ERAP2/ERAP1	1,035.1	**−**6.7	12	32

Buried surface area (BSA), binding affinity given as free energy of binding (Δ*G* ^est^) and the corresponding dissociation constant (*K*_d_ at 25°C), and number of intermolecular (IM) contacts were calculated using the initial, energy-minimized structures.

**Table 2:** Summary of mean buried surface area (BSA) and mean estimated binding free energy (Δ*G* ^est^), calculated from triplicate 100–ns MD simulations of each system (designated as in **Table 1**).

System topology	BSA (Å^2^)			Δ*G* ^est^ (kcal/mol)		
	MD–1	MD–2	MD–3	MD–1	MD–2	MD–3
**ERAP2 (A)**	**1306**	**1349**	**1177**	**–9.3**	**–9.4**	**–9.2**
ERAP1/ERAP2 (A1)	1135	1095	1126	–8.5	–8.5	–8.7
ERAP2/ERAP1 (A2)	1105	1020	1119	–8.6	–8.5	–8.6
**ERAP2 (B)**	**1607**	**1692**	**1608**	**–11.5**	**–12.1**	**–11.9**
ERAP1/ERAP2 (B1)	1348	1339	1517	–7.4	–8.0	–8.3
ERAP2/ERAP1 (B2)	1675	1761	1680	–10.4	–10.7	–10.6
**IRAP (C)**	**2624**	**2589**	**2576**	**–11.1**	**–10.9**	**–11.0**
ERAP1/ERAP2 (C1)	1546	1421	1118	–7.2	–7.6	–7.0
ERAP2/ERAP1 (C2)	1313	1833	1088	–7.6	–8.0	–7.3
**APN (D)**	**1981**	**2064**	**1978**	**–9.6**	**–9.5**	**–9.6**
ERAP1/ERAP2 (D1)	1349	1399	1473	–7.9	–8.0	–8.0
ERAP2/ERAP1 (D2)	877	414	565	–6.8	–5.4	–6.4

**Table 3:** Summary of mean buried surface area (BSA) and mean estimated binding free energy (Δ*G* ^est^), calculated from triplicate 100–ns MD simulations of ERAP1–c-Jun/ERAP2–c-Fos starting from the preferred ERAP2–based topology (model B2) and in comparison with an IRAP–based topology (model C2).

ERAP2–c–Fos / ERAP1–c–Jun	BSA (Å^2^)			Δ*G^est^* (kcal/mol)		
	MD1	MD2	MD3	MD1	MD2	MD3
Model B2-based	5225 (2667)	4634 (2260)	4217 (1704)	–18.8	–17.1	–16.2
Model C2-based	4436 (1597)	4652 (2018)	4698 (2196)	–13.5	–13.9	–13.9

Values in parentheses indicate the BSA of residues in the ERAP1/ERAP2 interface.

## Results and Discussion

### Heterodimeric Models Based on ERAP2

With the aim to produce meaningful initial models of a putative ERAP1/ERAP2 heterodimer, we first employed the crystallographic structures of ERAP2. This was the obvious choice given the high sequence and structural homology of ERAP1 and ERAP2 (49% sequence identity) and the wealth of available X-ray structures of ERAP2. To date, 9 crystal structures of ERAP2 have been resolved at a resolution range of 2.5–3.2 Å, either in complex with lysine [PDB IDs: 3se6 (7), 4e36 (43)], or several other peptidomimetic [4jbs (44), 5ab0 (13), 5ab2 (13)] and small-molecule inhibitors [5j6s (45), 5k1v (45), 6ea4^1^], including ERAP2 in the apo form [5cu5 (13)]. In all these crystal structures, the asymmetric crystallographic unit comprised of an ERAP2 homodimer in which dimerization was facilitated mainly through the N-terminal domain I and to a lesser extent by the C-terminal domain IV (**Supplementary Figure S2**). In the first X-ray structure of ERAP2 (PDB IDs: 3se6) (7) the total buried surface area is 1150 Å^2^, of which 970 Å^2^ is from domain I and 160 Å^2^ from domain IV. In this arrangement, the hinge region of ERAP2 in domain III does not participate in dimer formation, whereas the two catalytic sites in domain II face each other, which was hypothesized to facilitate exchange of the peptide substrates during catalytic cycles (7). Considering that most of the residues that participate in ERAP2 dimerization are conserved in ERAP1, the authors proposed that the crystallographic homodimer of ERAP2 is a suitable model for the ERAP1/ERAP2 heterodimer (7).

A subsequent X-ray structure of ERAP2 in complex with a potent phosphinic inhibitor (a transition state analogue) (13) revealed a region of ERAP2 that was not visible in the earlier and lower-resolution structures. This 23-residue sequence is encoded by exon 10 of the gene and has been implicated in the retention mechanism of ERAP1 through a disulfide bond formation with the ER-resident disulfide isomerase Erp44 (46). Similarly, in most crystal structures of ERAP1 the corresponding region is not visible. In fact, in the construct employed in the high-resolution ERAP1 structure used here (PDB ID: 6q4r) (28), a 28-amino acid sequence (residues 486−513) that is encoded by exon 10 and part of exon 11 of the ERAP1 gene, were substituted by a short loop consisting of a –GSG– sequence. In this particular ERAP2 structure however, the exon 10 loop formed an appendix extending from helix 8 of domain II and was stabilized through a disulfide bond between Cys503 and Cys514. Interestingly, this loop was also stabilized in the crystal by a symmetry-related monomer (**Supplementary Figure S3**), mainly through insertion of Met520 into a hydrophobic pocket of the adjacent ERAP2 molecule. This interaction had not been observed in any other crystal structure of ERAP1 or ERAP2 and has been proposed to be a structural template that facilitates interactions between the two enzymes and presumably other proteins in the ER, so as to regulate retention and extracellular function in the context of blood pressure regulation or innate immune responses (46, 47). Although the exon 10 sequence is not conserved in ERAP1 (**Supplementary Figure S3**), the positions of these two cysteine residues are highly similar, suggesting that a similar disulfide bond may be forming in ERAP1. The structural integrity of this solvent-accessible disulfide bond that stabilizes the exon 10 loop is possibly regulated by the local redox potential, or interactions with disulfide isomerases.

**Figure 1:**
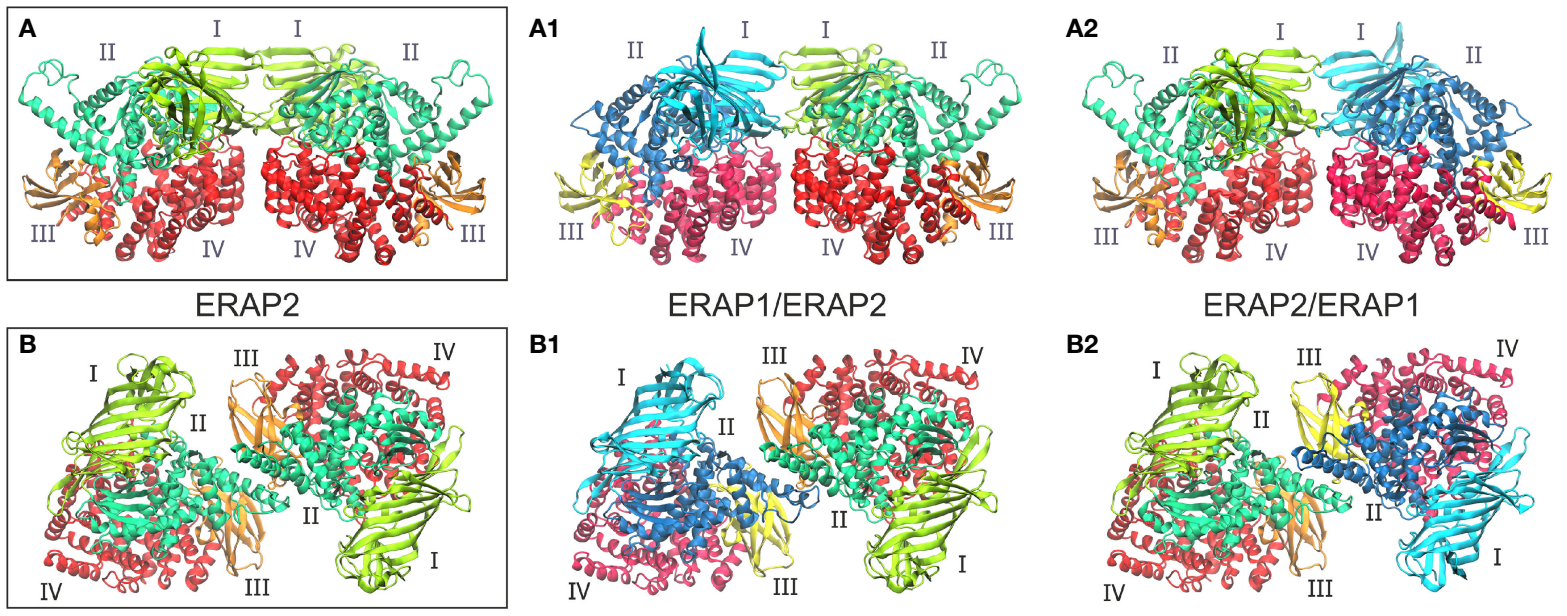
**(A, B)** Crystallographic structures of ERAP2 homodimers in the two different topologies formed within the unit cell, and which were used as templates for the 4 heterodimeric ERAP1/ERAP2 models. For each dimer, both possible topologies of ERAP1/ERAP2 (A1, B1) and ERAP2/ERAP1 (A2, B2) heterodimers were investigated. Domains are color-coded as indicated for each enzyme with Latin numbers.

Taken together, these observations led us to employ both experimentally observed ERAP2 homodimeric topologies as templates for the generation of heterodimeric ERAP1/ERAP2 models. The first set of models is based on the dimerization topology as observed in the asymmetric unit (hereafter designated as model A) in the two possible arrangements obtained by superimposing ERAP1 on either chain (designated as models A1 and A2, **Figure 1**). The second set of models is based on the ERAP2 dimerization topology mediated by the symmetry-related molecule along the –z axis (model B), again in both possible arrangements (B1 and B2, **Figure 1**). To construct these models, we employed coordinates of the highest resolution crystal structures of ERAP1 (PDB ID: 6q4r, resolved at 1.6 Å) (28) and ERAP2 (PDB ID: 5ab0, resolved at 2.5 Å). However, considering that the dimerization interface of the heterodimeric models B1 and B2 comprise the exon 10 loop (**Supplementary Figure S3**), we modeled this region of ERAP1 using the corresponding residues resolved in ERAP2 as a template (Cys486 and Cys498 of ERAP1 were also linked *via* a disulfide bridge).

### Heterodimeric Models Based on Other M1 Aminopeptidases

Although size-exclusion chromatography and dynamic light scattering analyses indicate that ERAP2 is predominantly monomeric in solution at concentrations below 1 mg/ml, another member of the M1 aminopeptidases, IRAP, has been found to be fully in a dimeric state, both in solution and in the crystal structures determined so far (48–50) In contrast to ERAP2 that has been shown to form crystallographic dimers through the N-terminal domain (**Supplementary Figure S2**), IRAP was found to form dimers through its C-terminal domain (**Supplementary Figure S4**) (49, 50). Interestingly, this dimerization topology is similar to the homologous aminopeptidase N (APN) (30) and aminopeptidase A (APA) (51), both of which form functional dimers. Interestingly, although IRAP, APN and APA form homodimers, different regions of their C-terminal domains are involved in dimer formation, leading to completely different dimer interfaces. This arrangement allows for the closed-to-open conformational changes, during which the dimer interfaces are maintained and the membrane anchors move laterally in the cell membrane.

**Figure 2:**
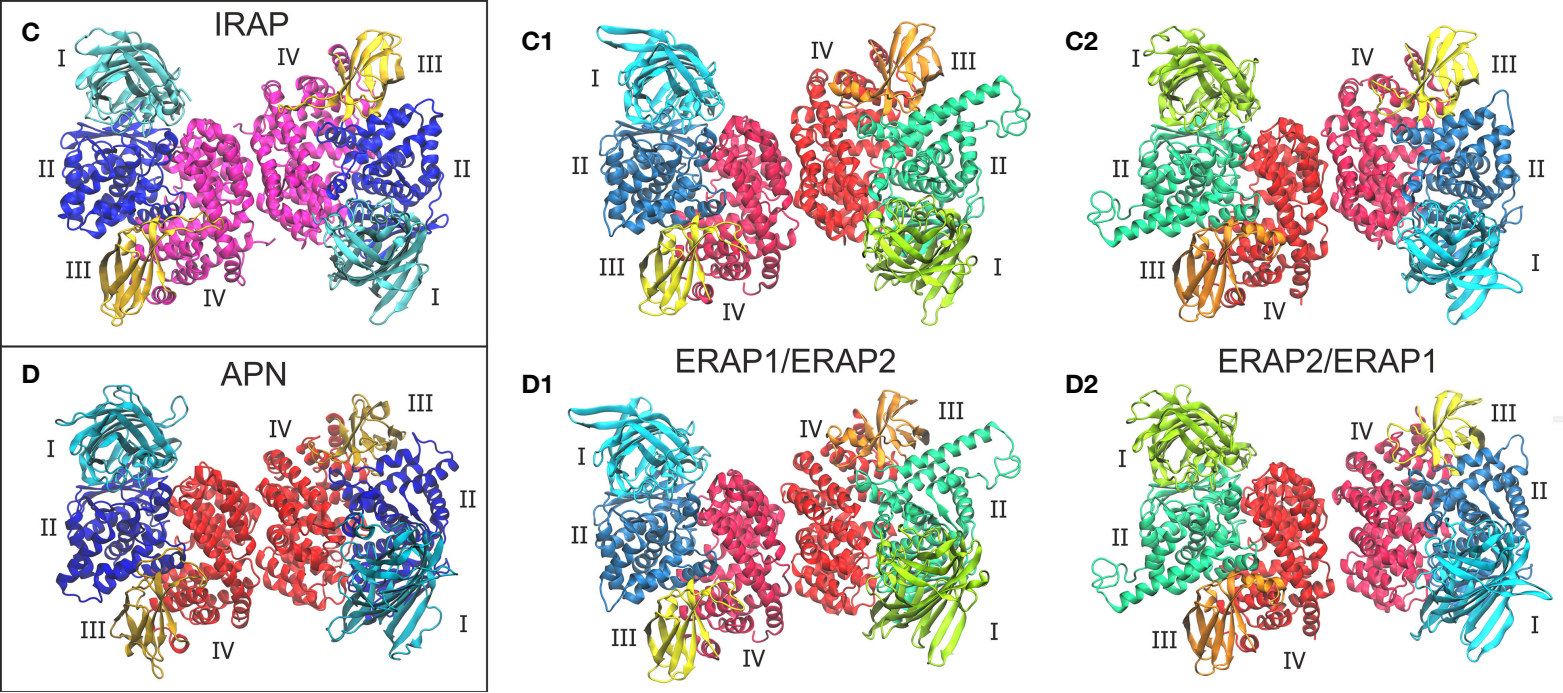
Crystallographic structures of the homodimeric IRAP **(C)** and APN **(D)**, which were employed as templates for the construction of the corresponding ERAP1/ERAP2 (C1, C2) and ERAP2/ERAP1 (D1, D2) heterodimeric topologies. Domains are color-coded as indicated for each enzyme with Latin numbers.

Given the high structural and sequence homology of IRAP with ERAP1 (48% sequence identity) and ERAP2 (45% sequence identity), we sought to investigate the potential that ERAP1 and ERAP2 can form dimers with a similar topology as the IRAP homodimer. On this basis, we employed the highest-resolution X-ray crystal structure of IRAP (PDB ID: 5mj6 resolved at 2.5 Å) (29) as another template for the construction of two putative ERAP1/ERAP2 topologies (models C1 and C2, **Figure 2**). For comparison, we also employed the high-resolution crystal structure of human APN (PDB ID: 4fyt resolved at 1.8 Å) (30) as an additional template of the two heterodimeric models (models D1 and D2, **Figure 2**). The inhibitors resolved in the X-ray structures of IRAP (DG026, a phosphinic-based tripeptide) and APN (amastatin, a hydroxamic-acid based inhibitor) have been substituted with the phosphinic dipeptide (PSE) that was used in all ERAP2-based dimers. Similarly, all glycan moieties resolved in the crystal structures have been retained.

A summary of the crystallographic homodimers used as templates (models A–D) and the corresponding ERAP1/ERAP2 heterodimeric models is given in **Table 1**. The buried surface area (BSA) was calculated using the MSMS module of VMD, whereas the estimated binding affinity, dissociation constant *K*_d_ at 25°C and number of intermolecular contacts were calculated using PRODIGY (42, 52). As evident from these data, IRAP displays the highest BSA and number of intermolecular (IM) contacts, followed by APN and the symmetry-related ERAP2 (model B) homodimer. It should be noted that the empirically-based equation employed by PRODIGY for the prediction of Δ*G* is based on a simple linear regression of the number of interacting residues at the interface (IC: interface contacts), including terms with properties of the non-interacting surfaces (NIS) that have been shown to play important role in binding affinity, especially with polar and charged residues (53). Therefore, favorable long-range electrostatic interactions and preferential solvation of non-interacting dimeric surface may account for the relatively high Δ*G* ^est^ of model B (ERAP2), albeit the significantly lower BSA with respect to model C (IRAP).

Interestingly, this analysis indicates that if ERAP2 associates in a dimeric form, then model B is a more preferable topology with respect to model A, as it exhibits a higher number of contacts and a larger buried surface area (**Table 1**). With regard to the potential heterodimeric complex of ERAP1/ERAP2, the preferred topology based on the estimated binding affinity is model B2. However, model D1 (based on APN) exhibits larger BSA and significantly more contacts.

### Molecular Dynamics Simulations

To further optimize and evaluate the generated ERAP1/ERAP2 models, we investigated the structural dynamics of the 8 heterodimeric systems by means of classical, all-atom molecular dynamics (MD) simulations. For comparison, the 4 homodimeric models were also investigated through unrestraint MDs in explicit solvent. Three independent MD simulations of 100 ns were performed after equilibration of 10 ns for each system, to allow re-orientation of the dimers within aqueous environment and obtain statistical information for the measured values.

Examination of the root–mean–square deviations (RMSD) of the protein backbone from the initial model was used to investigate the structural rearrangement of the complexes (**Supplementary Figures S5**, **S7**). From these plots it is evident that the two functional homodimers of IRAP and APN displayed the lowest RMSD from the initial topology with respect to the two homodimeric topologies of ERAP2 (**Supplementary Figure S5**). Given that both IRAP and APN in our simulation systems lack the anchoring N-terminal transmembrane domain, this observation indicates that their crystallographic homodimers display an optimal topology of their interface in solution too. Regarding the ERAP1/ERAP2 heterodimer, most of the models displayed high RMSD from the initial topology (**Supplementary Figure S7**), indicating significant rearrangement of the complexes. The lowest deviations were exhibited by models A1 and A2, whereas model D2 displayed the highest deviations, which is in accordance with the initial low propensity of this topology for a favorable dimerization (**Table 1**). It should be noted however, that the structure of the monomers does not deviate significantly from the initial, crystallographic closed state of each enzyme (**Supplementary Figures S6**, **S8**). In most simulations of the 4 homodimeric models, each protomer does not deviate more than 2.5 Å (RMSD of all C^α^ atoms) from the initial structure, with only a few exceptions (**Supplementary Figure S6**). In the simulations of the 8 heterodimers, ERAP1 displays typical RMSD values below 2 Å with respect to the X-ray structure, whereas ERAP2 structures displayed higher deviations in most of the simulations. Still, RMSD values below 3 Å when fitted with respect to the initial structure do not indicate major conformational changes (**Supplementary Figure S8**).

The high deviations from the initial topology observed in the simulations of many heterodimeric complexes can be interpreted as an indication of low stability. However, the possibility that the complex rearranges during the MD simulations towards higher affinity states in order to maximize the dimeric interface cannot be excluded. For this reason, we extracted 100 equally-sampled snapshots from the three 100-ns productions runs of each system, which were used to calculate the buried surface area (BSA) and the binding affinity (Δ*G* ^est^) of each dimer. The resulting plots of BSA as a function of simulation time for the homodimers (**Supplementary Figure S9**) and for the heterodimers (**Supplementary Figure S10**) are in accordance with the observations discussed above. Thus, the homodimer complexes exhibited the lowest fluctuations as a function of time with small deviations from the initial dimer. Examination of the mean values obtained for the 4 homodimers (**Table 2**) are in good agreement with the initial observations using the energy-minimized crystallographic models (**Table 1**). IRAP exhibits the most stable homodimer with a mean BSA = 2596 ± 25 Å^2^ and an estimated Δ*G* = **−**11.0 ± 0.1 kcal/mol (standard deviation from N = 3 simulations), followed by APN homodimer that exhibits mean BSA = 2,008 ± 49 Å^2^ and an estimated Δ*G* = −9.6 ± 0.1 kcal/mol. Regarding the two ERAP2-based homodimeric models, the ERAP2 homodimer from the asymmetric unit (model A) displayed a mean BSA = 1,277 ± 89 Å^2^ and an estimated Δ*G* = **−**9.3 ± 0.1 kcal/mol, whereas ERAP2 dimer formed by a symmetry-related molecule (model B), displayed a mean BSA = 1,636 ± 49 Å^2^ with an estimated Δ*G* = −11.8 ± 0.3 kcal/mol.

**Figure 3:**
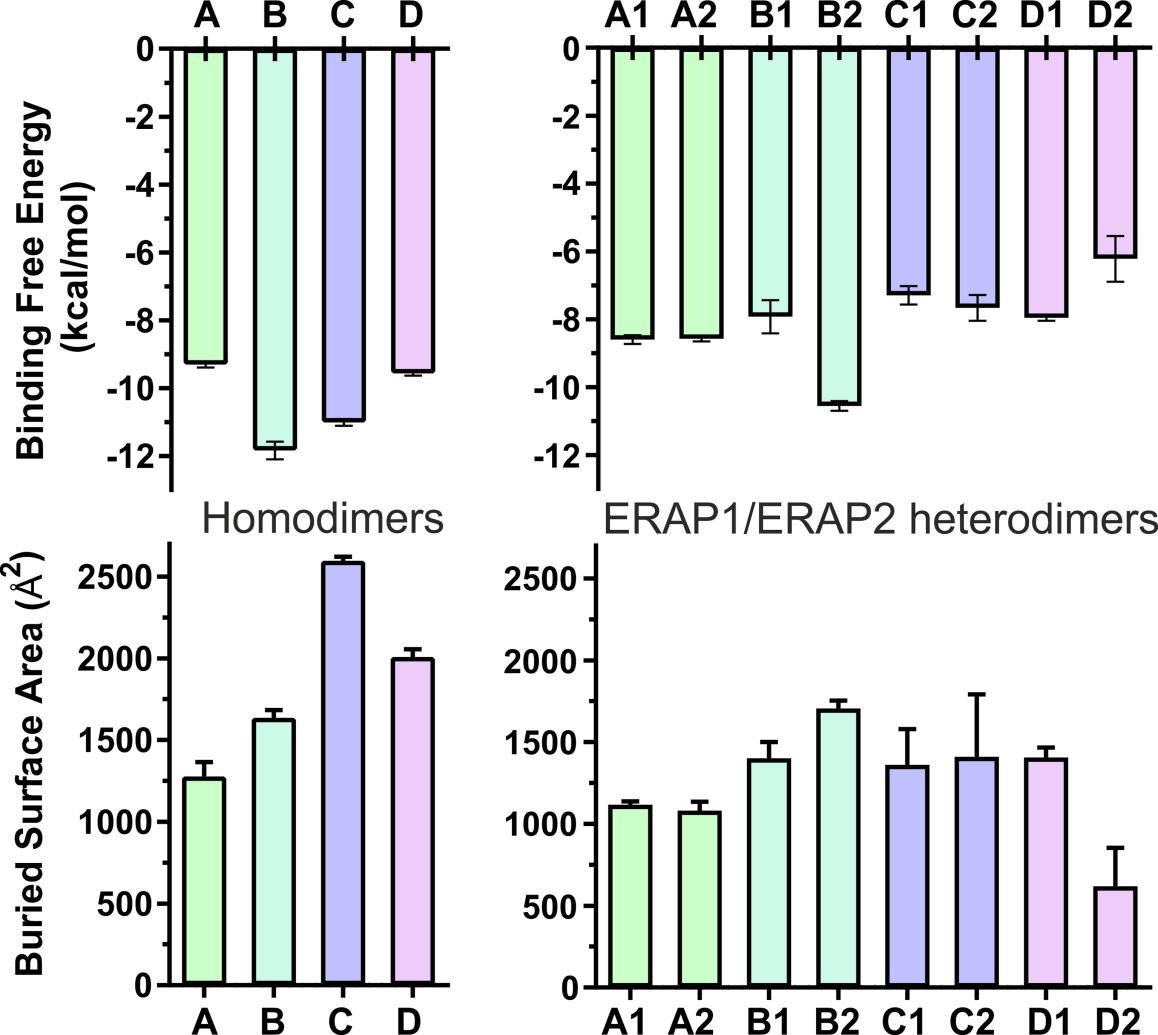
Bar plots of the estimated binding affinity (Δ*G* ^est^, upper panels) and the buried surface area (BSA, lower panels) for each crystallographic homodimer (left panels) and the corresponding heterodimeric models of ERAP1/ERAP2 (right panels). Mean values were calculated from 3 independent 100-ns MD simulations, and error bars indicate standard deviation.

These results lead us to propose that the interaction of ERAP2 domains II, including the exon 10 loop, in model B is likely stronger than the interactions of domains I displayed by the homodimeric ERAP2 model A. Unexpectedly, the estimated binding affinity of model B is equally, if not higher, than that estimated affinity for the physiologically relevant IRAP homodimer in solution (**Figure 3**). This constitutes an apparent contradiction to the lower BSA calculated for ERAP2 model B in comparison with the BSA of IRAP and even that of APN. The reason is probably due to the empirically-based equation employed by PRODIGY for the prediction of Δ*G* (see *Materials and Methods* section), which is based on a simple linear regression of the number of interacting residues at the interface (IC: interface contacts), including terms with properties of the non-interacting surfaces (NIS) that have been shown to play important role in the binding affinity, especially with polar and charged residues (53). Therefore, regulation of protein–protein interaction through long-range electrostatics and preferential solvation of non-interacting dimeric surface may account for the high estimated Δ*G* of model B that display lower BSA than that of IRAP (model C). On the other hand, considering the root mean square error of 1.9 kcal/mol for the predicted binding affinities employed during development of the method, a difference of Δ(Δ*G*) = 2 kcal/mol should be considered tentative.

**Figure 4:**
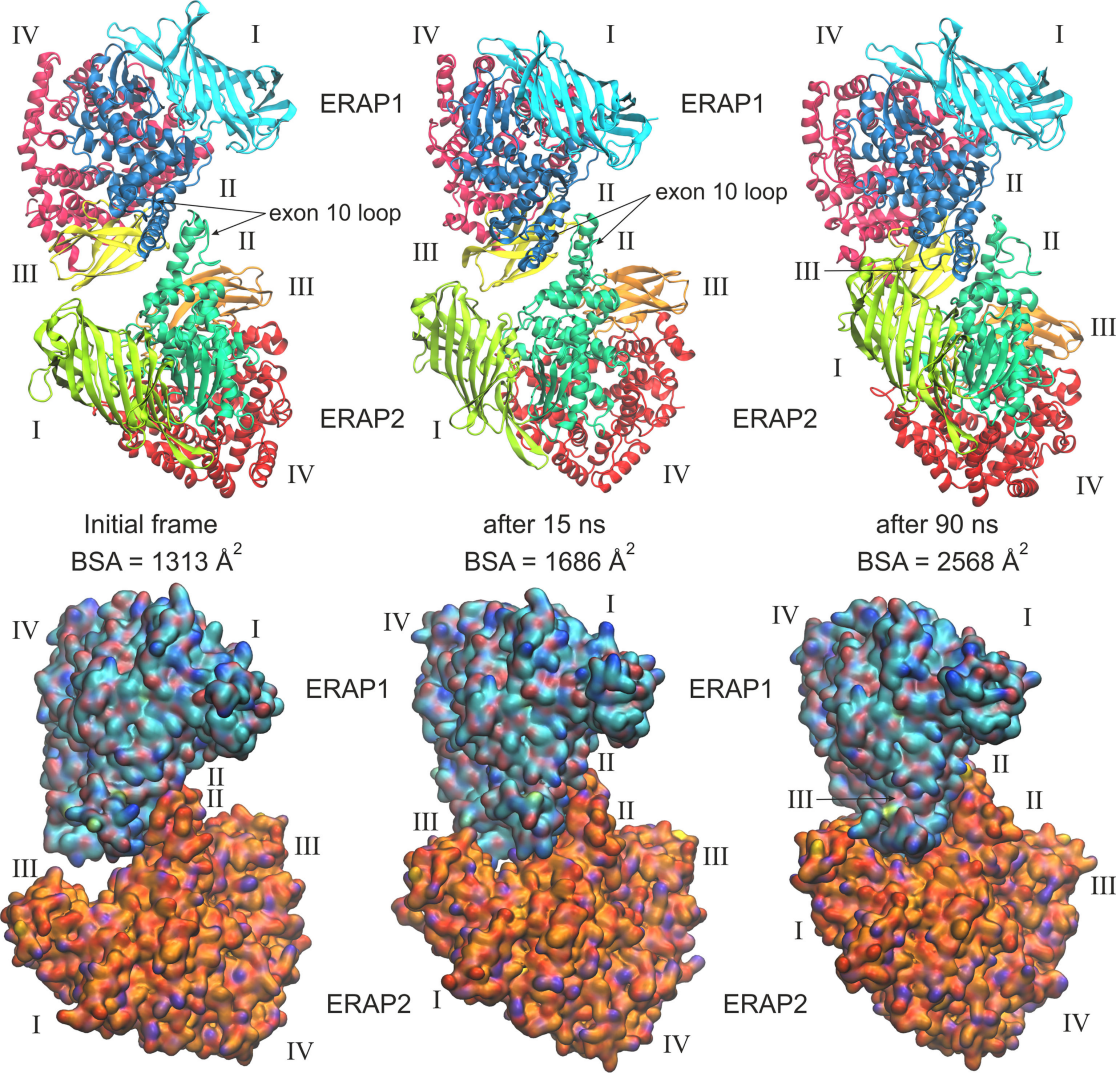
Ribbon representations (upper panels) and surface representations (lower panels) of 3 frames from one MD simulation of the most favorable heterodimeric ERAP1/ERAP2 topology B2, illustrating the initial, energy minimized and two snapshots at 15 and 90 ns of the simulation. Domains are designated with Latin numbers and surfaces are colored with cyan C atoms for ERAP1 and orange C atoms for ERAP2. The position of the interacting exon 10 loop and the buried surface area (BSA) of each complex are indicated.

When considering the heterodimeric models, our results reveal that model B2 is the most preferred topology of the ERAP1/ERAP2 complex **(** **Figure 3**). This complex displayed a mean BSA of 1,705 ± 48 Å^2^ with a mean estimated Δ*G* = −10.6 ± 0.1 kcal/mol from triplicate MDs. Although the mean BSA is lower than those displayed by the homodimers of IRAP and APN, the estimated binding affinity of model B2 is comparable with that of the two functional homodimers. Examination of the MD trajectories from the simulations of model B2 revealed that the dimer rearranges significantly with respect to the initial configuration (**Figure 4**). While the dimeric interface comprises residues from domains II of ERAP1 and ERAP2 in the initial model, domain III of ERAP1 and domain I of ERAP2 come into contact during the MD simulations. As a result, the BSA of the dimer increases as a function of simulation time (**Supplementary Figure S10**), giving rise to higher mean values with respect to the initial geometries. In contrast, the heterodimeric models based on the topology of IRAP and APN (models C1,2 and D1,2 respectively) that had high initial BSA, displayed rearrangements during the MD simulations that decreased the number of contacts and BSA, an effect that was more pronounced for models D1 and D2 (**Supplementary Figure S10**).

**Figure 5:**
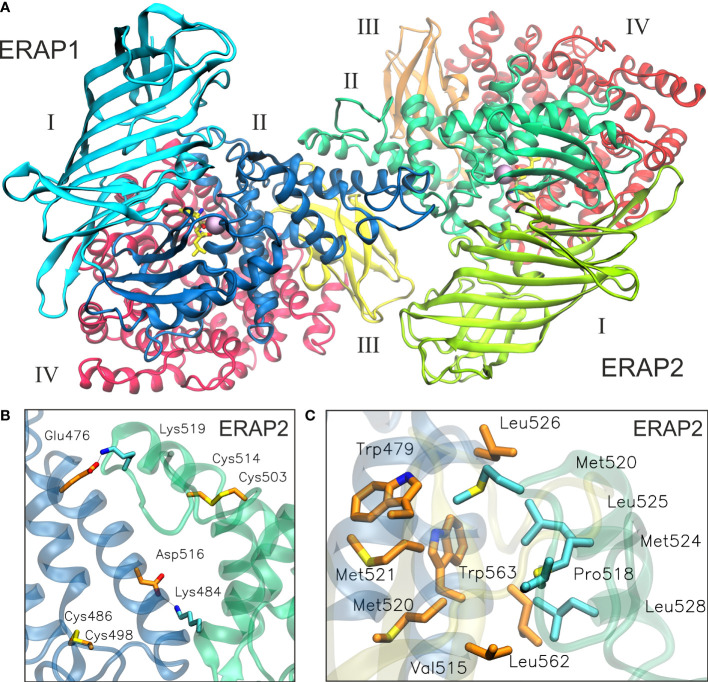
**(A)** The proposed heterodimeric ERAP2/ERAP1 model B2 from a representative snapshot taken from a 100-ns MD simulation. The snapshot is the centroid of the highest populated cluster of conformations that represents 64% of the trajectory within 2 Å RSMD of all Cα atoms. **(B)** Close-up view showing two key salt–bridge interactions between helices 8 of ERAP1 (orange C atoms) and ERAP2 (cyan C atoms). The disulfide bridges that stabilize exon 10 loops are shown as sticks. **(C)** Another close-up view of the dimeric interface illustrating hydrophobic/aromatic interactions between ERAP1 (orange C atoms) and ERAP2 (cyan C atoms).

Taken together, the results of our MD simulation suggest that the most favorable topology of ERAP1/ERAP2 heterodimer is model B2, in which the dimeric interface comprises mainly of residues from domain II of the enzymes (**Figure 5**, also provided as a PDB file in **Supplementary Material**). Although substrate access is possible in all models of the heterodimers employed in this study, it should be noted that an interaction between domains II and III of ERAP1 with domains I and II of ERAP2 is expected to affect the dynamics of their closed-to-open states (11). A closer look at the interface reveals key polar interactions, including that between Glu476 and Asp516 of ERAP1 with Lys484 and Lys519 of ERAP2, and also a patch of hydrophobic interactions between Trp479, Leu526, Leu562 and Trp563 of ERAP1 with Met520, Met524, Leu525 and Leu528 of ERAP2 (**Figures 5B, C** and **Supplementary Table S2**). In this topology, the presence of two disulfide bridges in the exon 10 loops of ERAP1 and ERAP2 could play a key role in the formation of the heterodimer, considering that their oxidation state is related to the stability of the highly flexible loop. Therefore, the local redox potential may be critical for the formation and stabilization of this heterodimeric complex.

### Model of ERAP1–Jun/ERAP2–Fos Heterodimer

**Figure 6:**
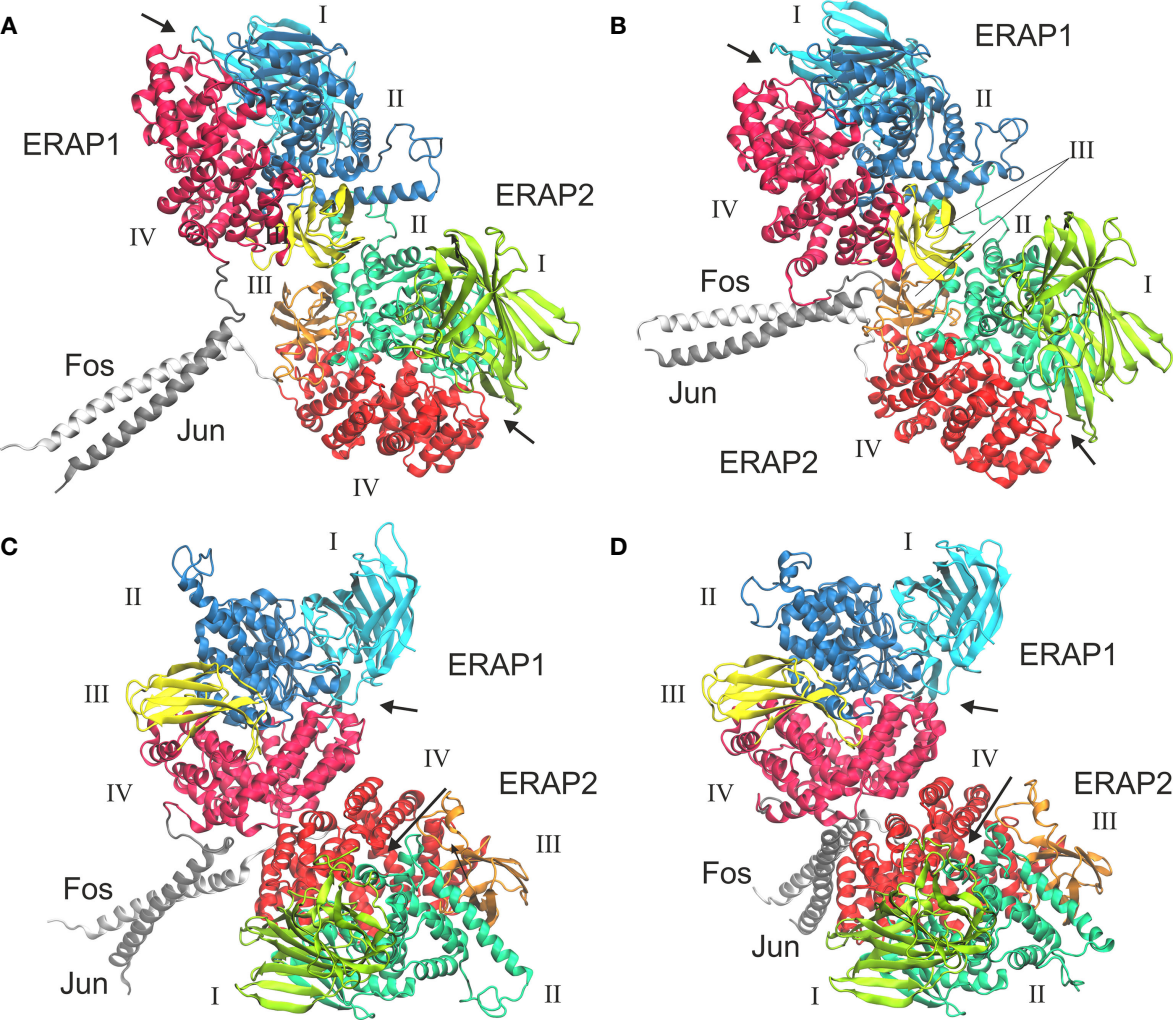
Models of ERAP1–c-Jun/ERAP2–c-Fos based on two different topologies. **(A)** Initial, energy minimized dimer based on model B2 and **(B)** representative structure from a MD simulation, calculated as the centroid of the highest populated cluster of conformations (40% fraction of the trajectory) with respect to the RMSD of the C^α^ atoms excluding the Jun/Fos linker residues. **(C)** Initial, energy minimized structure of the IRAP-based model C2, and **(D)** representative structure of the highest populated cluster of conformations (43% fraction of the trajectory) calculated from a MD simulation. Arrows indicate the substrate entrance site.

In view of the experimental c-Jun/c-Fos-stabilized ERAP1/ERAP2 construct (23), we asked whether the preferred model presented here is compatible with the experimental construct. For this reason, we prepared a model of ERAP1–c-Jun/ERAP2–c-Fos based on model B2 (**Figure 6A**) and subjected it to MD simulations as described for the other heterodimers. For comparison, we also employed a model of ERAP1–c-Jun/ERAP2–c-Fos based on model C2 (**Figure 6C**). From the initial construction of the models, it was evident that both topologies were possible, given the long linker employed between the C-termini of ERAP1/ERAP2 and the N-termini of Jun/Fos. Our MD simulations revealed that model B2 experienced higher structural deviation from the initial orientation with respect to model C2 (**Supplementary Figure S11**). As an effect, the ERAP1/ERAP2 complex increased the BSA as a function of simulation time in model B2 (**Figure 6B**), whereas model C2 (**Figure 6D**) displayed lower fluctuations and deviations from the initial topology (**Supplementary Figure S12**). Comparison of the mean BSA extracted from the 3 simulations reveals very similar values (4,692 ± 506 Å^2^ for B2 versus 4,595 ± 140 Å^2^ for C2), however, the B2 topology displayed significantly higher free energy of binding with a mean value of −17.4 ± 1.3 kcal/mol, versus −13.8 ± 1.3 kcal/mol for topology C2 (**Table 3**). Overall, our results demonstrate that the Jun/Fos-assisted ERAP1/ERAP2 heterodimerization is possible in either topology, with that of model B2 being the preferred arrangement in terms of binding affinity.

### Dimerization and ERAP1/ERp44 Interactions

In a previous study ERAP1 has been proposed to interact with the disulfide-shuffling chaperone ERp44 through the formation of disulfide bonds between Erp44 and the cysteines in the exon 10 loop (46). This mechanism was proposed to underlie the retainment of ERAP1 in the ER and its potential role in blood pressure regulation. If a possible ERAP1/ERAP2 dimerization utilizes the exon 10 loop, this would preclude Erp44 interactions. Furthermore, the two exon 10 loops in the dimer frequently approach each other during MD simulations, suggesting that the formation of inter-molecular disulfides is possible. Thus, it appears that such a dimerization topology would block ERAP1/Erp44 interactions. However, it is not known if all ERAP1 are constantly in complex with Erp44 and preliminary analysis suggests that the molar ratio of ERAP1 and ERAP2 in the ER is not 1:1 (16). Furthermore, the ERAP1/Erp44 dimerization has been suggested to be regulated by the redox potential in the ER. Thus, it is possible that both Erp44 and ERAP1 use a similar dimerization topology as ERAP2 and these interactions are transient regulatory components of an antigen processing machinery. Additional structural work will be necessary to fully understand the ERAP1/Erp44 interaction and its relationship to ERAP1/ERAP2 dimerization.

### Potency of Dimer Formation *In Vitro* and Inside the ER

To date, there has been no study demonstrating the isolation of a native ERAP1/ERAP2 dimer, in a form suitable for structural or biophysical studies. This contrasts with the IRAP homodimer that has been demonstrated to be stable in solution even at low concentrations (27). Our computational analysis suggests that even for the most favorable topology, the ERAP1/ERAP2 dimerization is energetically less favorable and would thus be weaker, something that would hinder *in vitro* isolation and characterization. In the crowded environment of the ER however, the conditions may resemble the ones found inside protein crystals and either in the presence or absence of adaptor proteins, weaker dimerization interactions could result in stable heterodimers. In this context, stabilization by means of an external bridging interaction such as the *fos/jun* dimerization domain described before (23) constitutes a reasonable solution, although it must be carefully engineered to avoid interference with enzyme mechanism and conformational changes. Our proposed model can be a useful starting point for optimizing a functionally intact ERAP1/ERAP2 complex for structural studies.

### Dimerization, Conformational Changes and Synergy

The function of ERAP1 and ERAP2 has been described as dependent on a large conformational change in which domain IV rotates to abut onto domain I/II using domain III as a hinge (11, 12). In our preferred dimerization topology, hinge domain III participates in interactions at the dimer interface something that could affect the dynamics of the conformational changes for both enzymes. This may underlie previous observations regarding ERAP1 and ERAP2 activity in the ERAP1/ERAP2 *fos/jun* dimer (23) and could constitute a mechanism for regulating the antigen processing activity in the ER. This model would maximize synergism opportunities between ERAP1 and ERAP2, beyond complementary specificities to direct regulation of each other’s activity depending on substrate processing stages.

### Implications of the Heterodimer Formation in Antigen Processing

The exact role of ERAP1/ERAP2 dimerization in the ER in antigen processing and presentation is still largely unknown and much needs to be done to clarify this interaction. Still, an “antigen processing complex” such as an ERAP1/ERAP2 dimer is conceptually appealing because it affords additional opportunities in both processing efficiency and in regulation of antigen trimming. Here, we explored the topology of such an interaction based on experimentally observed dimerization interfaces of ERAP2 or homologous enzymes. The proposed topology is consistent with a moderate dimer stability, which could be sufficient for the subcellular compartment where the enzymes reside or a transient/regulatory interaction. Furthermore, this topology is consistent with the known mechanism of action of the enzymes and could underlie synergism and cross-regulation. This structural template can be used to guide additional experimental work necessary to validate aspects of the ERAP1/ERAP2 interaction and its role in the generation of the immunopeptidome and adaptive immunity.

## Data Availability Statement

The original contributions presented in the study are included in the article/**Supplementary Material**. Further inquiries can be directed to the corresponding authors.

## Author Contributions

AP carried out the simulations and computational analysis and interpreted results. AM and ES analyzed and interpreted the results. All authors listed have made a substantial, direct, and intellectual contribution to the work and approved it for publication.

## Funding

This work has been funded by the Hellenic Foundation for Research and Innovation (HFRI) under the 1st call Support for Postdoctoral Researchers, project “ARIA”, grant number 303

## Conflict of Interest

The authors declare that the research was conducted in the absence of any commercial or financial relationships that could be construed as a potential conflict of interest.

## Publisher’s Note

All claims expressed in this article are solely those of the authors and do not necessarily represent those of their affiliated organizations, or those of the publisher, the editors and the reviewers. Any product that may be evaluated in this article, or claim that may be made by its manufacturer, is not guaranteed or endorsed by the publisher.

## Footnotes

1 Maben, Z., Stern, L.J. (structure released: 18-12-2019) *to be published.*
