# Supplementary material for: Can ERAP1 and ERAP2 Form Functional Heterodimers? A Structural Dynamics Investigation
Source: Front Immunol. 2022 Apr 20;13:863529. doi: 10.3389/fimmu.2022.863529 (PMC9065437; doi:10.3389/fimmu.2022.863529)
Supplement: Supplementary file 1 [file DataSheet_1.pdf]

Supplementary Material

Can ERAP1 and ERAP2 Form Functional Heterodimers? A Structural Dynamics Investigation

Athanasios Papakyriakou[1*], Anastasia Mpakali[1] and Efstratios Stratikos[1,2*]

[1]Insitute of Biosciences and Applications, National Centre for Scientific Research "Demokritos", Athens, Greece

[2]Laboratory of Biochemistry, Department of Chemistry, National and Kapodistrian University of Athens, Athens, Greece

*** Correspondence:**
Athanasios Papakyriakou, thpap@bio.demokritos.gr | Efstratios Stratikos, estratikos@chem.uoa.gr

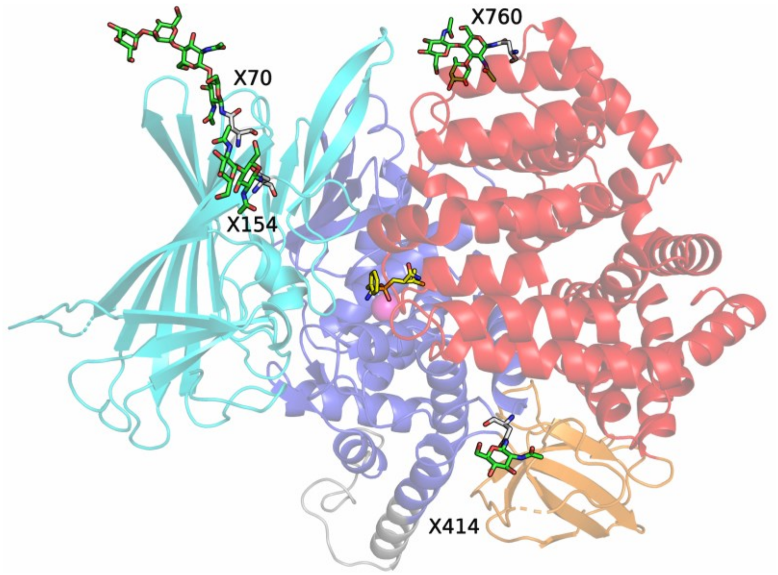

ERAP1	AMBER carbohydrate
Asn70	–4YB–4YB–3MB–0MA
Asn154	–4YB–0YB
Asn414	–0YB
Asn760	–WYB(–0fA)–0YB

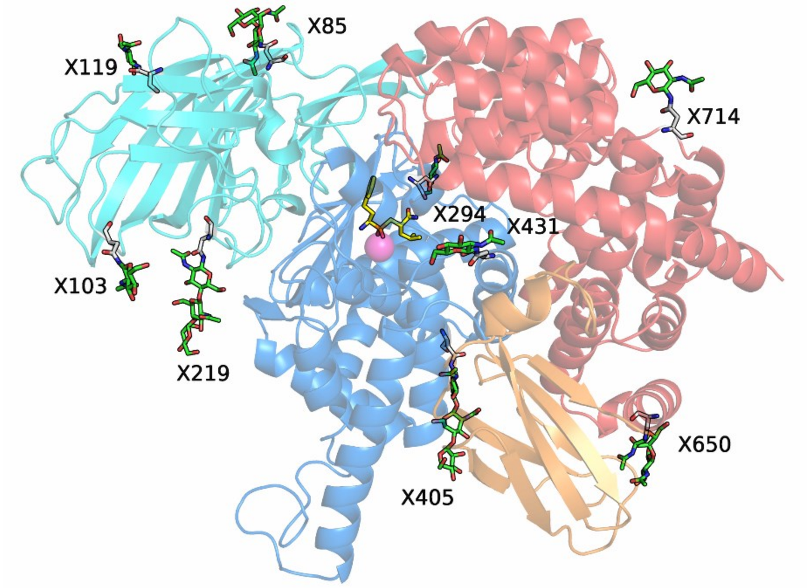

ERAP2	AMBER carbohydrate
Asn85	–4YB–0YB
Asn103	–0YB
Asn119	–0YB
Asn219	–4YB–4YB–0MB
Asn294	–0YB
Asn405	–4YB–4YA–0MA
Asn431	–0YB
Asn650	–4YB–0YB
Asn714	–0YB

Residue	Monosaccharide (linkage positions)
4YB	2-acetamido-2-deoxy-β-*D*-glucopyranose (4-)
4YA	2-acetamido-2-deoxy-α-*D*-glucopyranose (4-)
WYB	2-acetamido-2-deoxy-β-*D*-glucopyranose (3-, 4-)
0YB	2-acetamido-2-deoxy-β-*D*-glucopyranose
3MB	β-*D*-mannopyranose (3-)
0MB	β-*D*-mannopyranose
0MA	α-*D*-mannopyranose
0fA	6-deoxy-α-L-galactose

Figure S1. Cartoon representations of the enzyme structures used in this study, illustrating the position of sugar moieties with green-C sticks. The catalytic Zn(II) is shown as a magenta sphere with the bound PSE ligand in yellow-C sticks, while domains are color-coded as in the main text. For each glycosylation site, sugar residues are given with the AMBER GLYCAM06 code name for each monosaccharide.

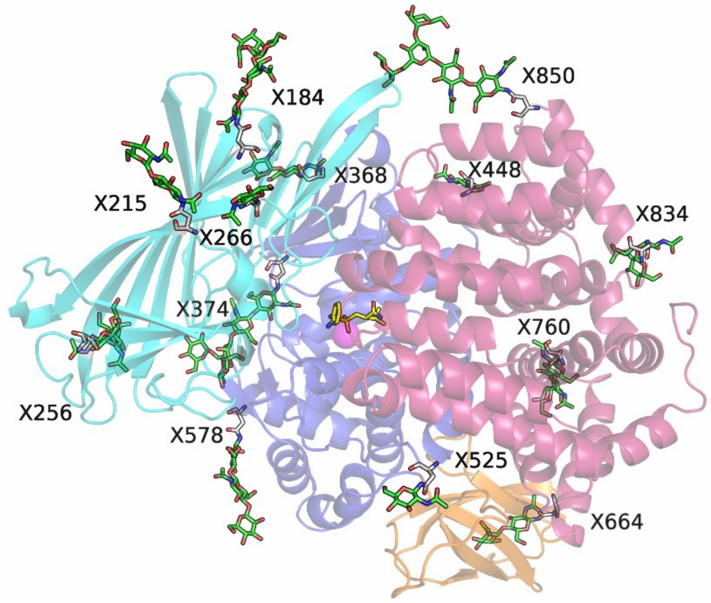

IRAP	AMBER carbohydrate
Asn184	–4YB–4YB–3MB–0MA
Asn215	–4YB–0YB
Asn256	–4YB–4YB–0MB
Asn266	–0YB
Asn368	–4YB–0YB
Asn374	–4YB–4YB–VMB(–0MA)–0MA
Asn448	–0YB
Asn525	–0YB
Asn578	–4YB–4YB–0YB
Asn664	–4YB–0YB
Asn760	–4YB–4YB–0MB
Asn834	–4YB–0YB
Asn850	–4YB–4YB–VMB(–0MA)–0MA

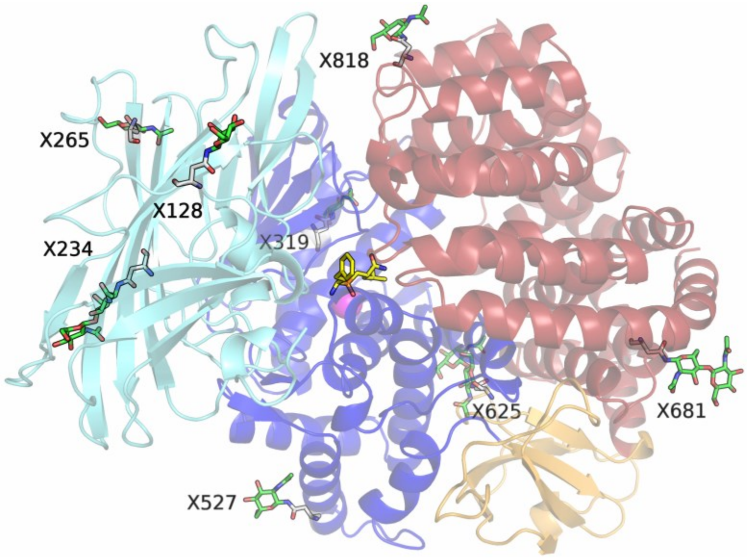

APN	AMBER carbohydrate
Asn128	–0YB
Asn234	–4YB–0YB
Asn265	–0YB
Asn319	–0YB
Asn527	–0YB
Asn625	–4YB–0YB
Asn681	–4YB–0YB
Asn818	–0YB

Residue	Monosaccharide (linkage positions)
4YB	2-acetamido-2-deoxy-β-*D*-glucopyranose (4-)
4YA	2-acetamido-2-deoxy-α-*D*-glucopyranose (4-)
0YB	2-acetamido-2-deoxy-β-*D*-glucopyranose
3MB	β-*D*-mannopyranose (3-)
VMB	β-*D*-mannopyranose (3-, 6-)
0MB	β-*D*-mannopyranose
0MA	α-*D*-mannopyranose

Figure S1 (cont.) Cartoon representations of the enzyme structures used in this study, illustrating the position of sugar moieties with green-C sticks. The catalytic Zn(II) is shown as a magenta sphere with the bound ligand in yellow-C sticks, while domains are color-coded as in the main text. For each glycosylation site, sugar residues are given with the AMBER GLYCAM06 code name for each monosaccharide.

Table S1. Sequence of the constructs employed for modeling of the ERAP1–Jun/ERAP2–Fos heterodimers, including the corresponding UniProt ID and residue range. The N-termini of ERAP1 and ERAP2 that are shown in grey were not modelled, while the short linkers are shown in red. Residues in bold indicate residues from crystallographic structures which were used as templates.

Protein construct	UniProt ID Residues	Sequence<br>1--50
**ERAP1**<br><br><br><br><br><br><br><br><br><br><br><br><br><br><br><br><br><br>**c-Jun**	**Q9NZ08-1**<br>46-941<br><br><br><br><br><br><br><br><br><br><br><br><br><br><br><br><br>**P05412-1**<br>276-315	MVFLPLKWSLATMSFLLSSLLALLTVSTPSWCQSTEASPKRSDGT**PFPWN**<br>**KIRLPEYVIPVHYDLLIHANLTTLTFWGTTKVEITASQPTSTIILHSHHL**<br>**QISRATLRKGAGERLSEEPLQVLEHPRQEQIALLAPEPLLVGLPYTVVIH**<br>**YAGNLSETFHGFYKSTYRTKEGELRILASTQFEPTAARMAFPCFDEPAFK**<br>**ASFSIKIRREPRHLAISNMPLVKSVTVAEGLIEDHFDVTVKMSTYLVAFI**<br>**ISDFESVSKITKSGVKVSVYAVPDKINQADYALDAAVTLLEFYEDYFSIP**<br>**YPLPKQDLAAIPDFQSGAMENWGLTTYRESALLFDAEKSSASSKLGITMT**<br>**VAHELAHQWFGNLVTMEWWNDLWLNEGFAKFMEFVSVSVTHPELKVGDYF**<br>**FGKCFDAMEVDALNSSHPVSTPVENPAQIREMFDDVSYDKGACILNMLRE**<br>**YLSADAFKSGIVQYLQKHSYKNTKNEDLWDSMASICPTDGVKGMDGFCSR**<br>**SQHSSSSSHWHQEGVDVKTMMNTWTLQKGFPLITITVRGRNVHMKQEHYM**<br>**KGSDGAPDTGYLWHVPLTFITSKSDMVHRFLLKTKTDVLILPEEVEWIKF**<br>**NVGMNGYYIVHYEDDGWDSLTGLLKGTHTAVSSNDRASLINNAFQLVSIG**<br>**KLSIEKALDLSLYLKHETEIMPVFQGLNELIPMYKLMEKRDMNEVETQFK**<br>**AFLIRLLRDLIDKQTWTDEGSVSERMLRSQLLLLACVHNYQPCVQRAEGY**<br>**FRKWKESNGNLSLPVDVTLAVFAVGAQSTEGWDFLYSKYQFSLSSTEKSQ**<br>**IEFALCRTQNKEKLQWLLDESFKGDKIKTQEFPQILTLIGRNPVGYPLAW**<br>**QFLRKNWNKLVQKFELGSSSIAHMVMGTTNQFSTRTRLEEVKGFFSSLKE**<br>**NGSQLRCVQQTIETIEENIGWMDKNFDKIRVWLQSEKL**ERMLEGGGSGG**R**<br>**IARLEEKVKTLKAQNSELASTANMLREQVAQLKQKVM**NHG
**ERAP2**<br><br><br><br><br><br><br><br><br><br><br><br><br><br><br><br><br><br>**c-Fos**	**Q6P179-1**<br>54-960<br><br><br><br><br><br><br><br><br><br><br><br><br><br><br><br><br>**P01100-1**<br>161-199	MFHSSAMVNSHRKPMFNIHRGFYCLTAILPQICICSQFSVPSSYHFTEDP<br>GAF**PVATNGERFPWQELRLPSVVIPLHYDLFVHPNLTSLDFVASEKIEVL**<br>**VSNATQFIILHSKDLEITNATLQSEEDSRYMKPGKELKVLSYPAHEQIAL**<br>**LVPEKLTPHLKYYVAMDFQAKLGDGFEGFYKSTYRTLGGETRILAVTDFE**<br>**PTQARMAFPCFDEPLFKANFSIKIRRESRHIALSNMPKVKTIELEGGLLE**<br>**DHFETTVKMSTYLVAYIVCDFHSLSGFTSSGVKVSIYASPDKRNQTHYAL**<br>**QASLKLLDFYEKYFDIYYPLSKLDLIAIPDFAPGAMENWGLITYRETSLL**<br>**FDPKTSSASDKLWVTRVIAHELAHQWFGNLVTMEWWNDIWLKEGFAKYME**<br>**LIAVNATYPELQFDDYFLNVCFEVITKDSLNSSRPISKPAETPTQIQEMF**<br>**DEVSYNKGACILNMLKDFLGEEKFQKGIIQYLKKFSYRNAKNDDLWSSLS**<br>**NSCLESDFTSGGVCHSDPKMTSNMLAFLGENAEVKEMMTTWTLQKGIPLL**<br>**VVKQDGCSLRLQQERFLQGVFQEDPEWRALQERYLWHIPLTYSTSSSNVI**<br>**HRHILKSKTDTLDLPEKTSWVKFNVDSNGYYIVHYEGHGWDQLITQLNQN**<br>**HTLLRPKDRVGLIHDVFQLVGAGRLTLDKALDMTYYLQHETSSPALLEGL**<br>**SYLESFYHMMDRRNISDISENLKRYLLQYFKPVIDRQSWSDKGSVWDRML**<br>**RSALLKLACDLNHAPCIQKAAELFSQWMESSGKLNIPTDVLKIVYSVGAQ**<br>**TTAGWNYLLEQYELSMSSAEQNKILYALSTSKHQEKLLKLIELGMEGKVI**<br>**KTQNLAALLHAIARRPKGQQLAWDFVRENWTHLLKKFDLGSYDIRMIISG**<br>**TTAHFSSKDKLQEVKLFFESLEAQGSHLDIFQTVLETITKNIKWLEKNLP**<br>**TLRTWLMVNT**LEGGGSGG**LTDTLQAETDQLEDEKSALQTEIANLLKEKEK**<br>**LEFILA**ASG

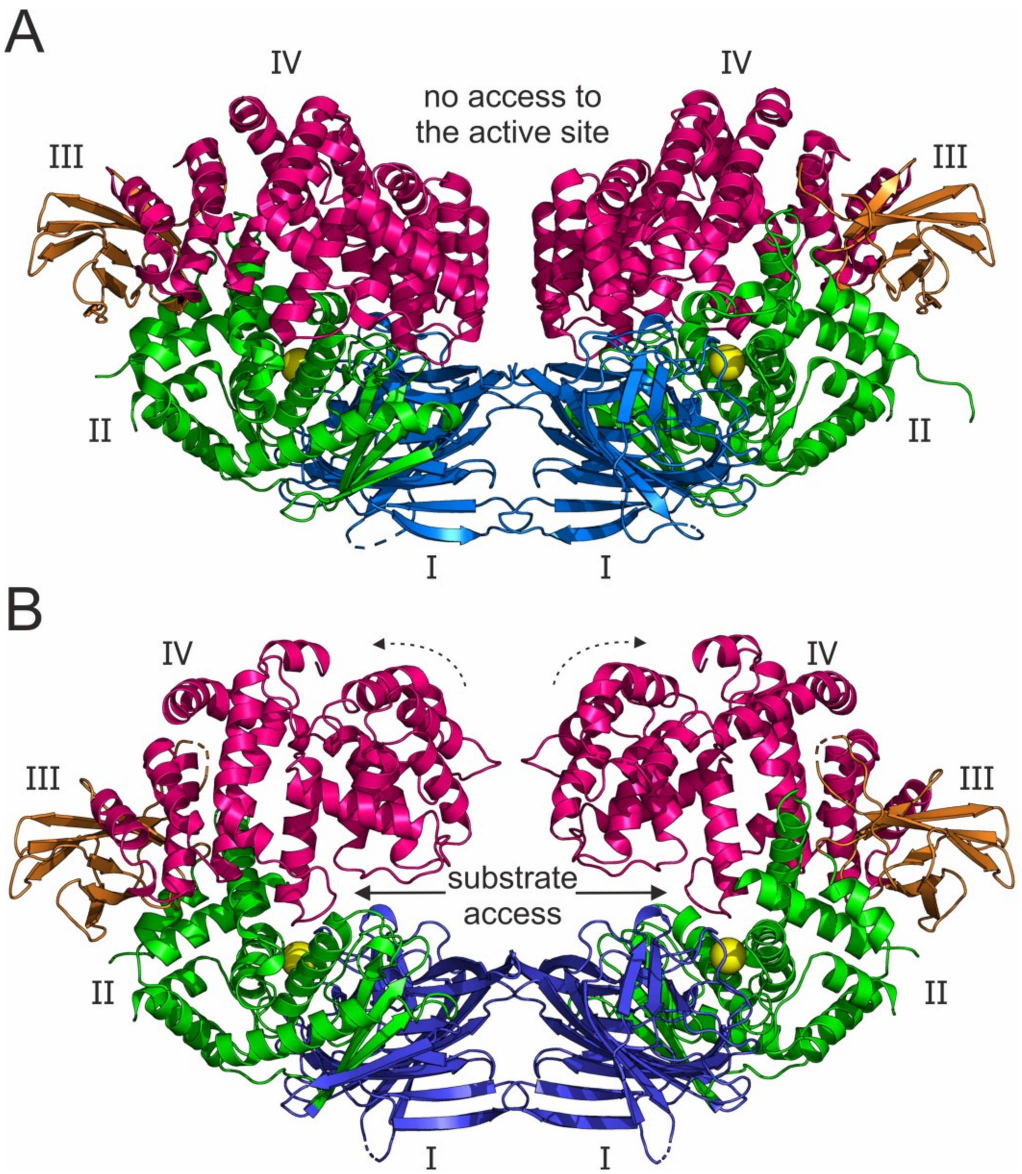

Figure S2. Proposed models of a ERAP1/ERAP2 heterodimer based on the first ERAP2 crystal structure (PDB ID: 3se6) illustrating the closed and open states of the enzymes. Their domains are color-coded as indicated by Latin numbers and the active site Zn(II) atom is shown as yellow sphere.

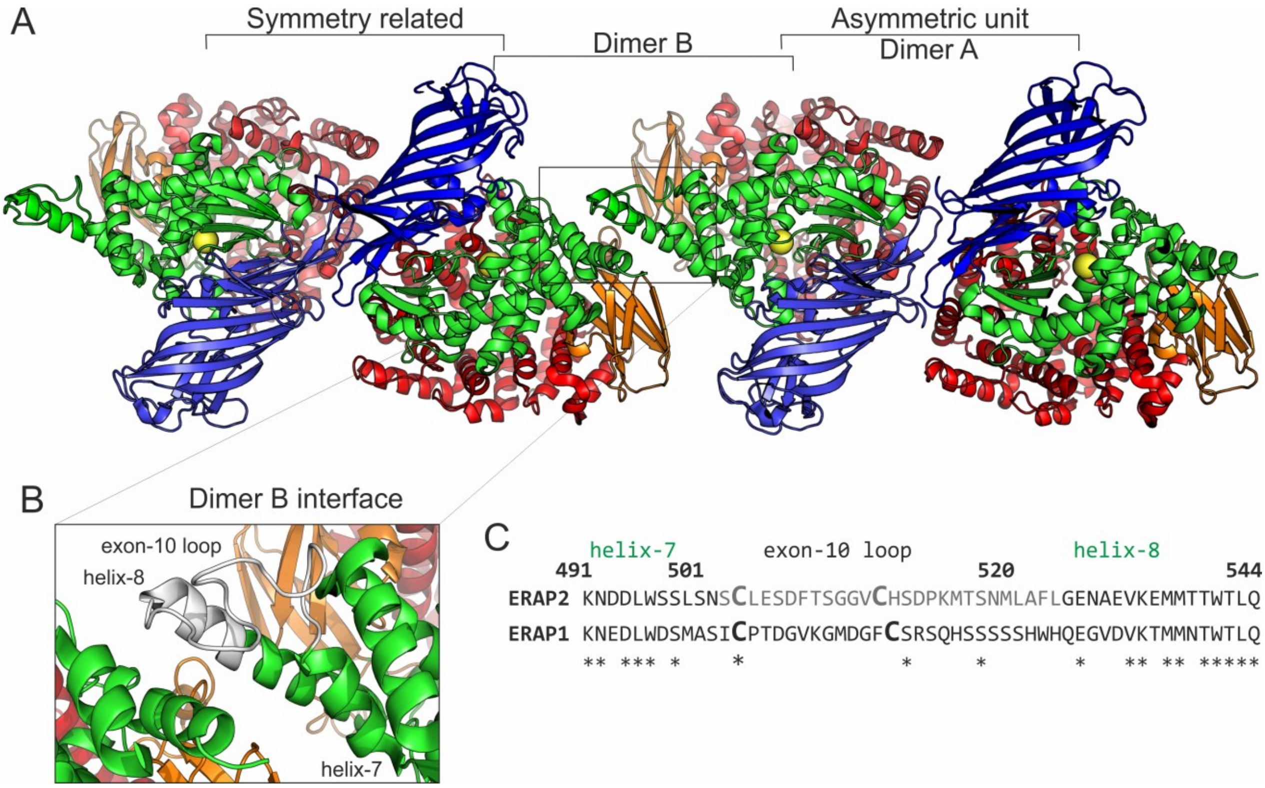

Figure S3. (A) View of the crystallographic ERAP2 homodimers as determined in complex with the decapeptide phosphinic inhibitor DG025 (PDB ID: 5ab0), illustrating the two molecules of the asymmetric unit (dimer A) and a symmetry-related unit along the –z axis. The dimer formed between chain A of the asymmetric unit and chain B of the symmetry related unit is designated as dimer B. Domains are color-coded as in Figure S2. (B) Close-up view of the contact region in dimer B, highlighting the exon-10 loop and the helix-8 region that was not resolved in the first X-ray structure of ERAP2 (PDB ID: 3se6). (C) Sequence alignment showing the lack of conservation in the exon-10 loop of ERAP2 and ERAP1, while the two conserved cysteine residues are highlighted.

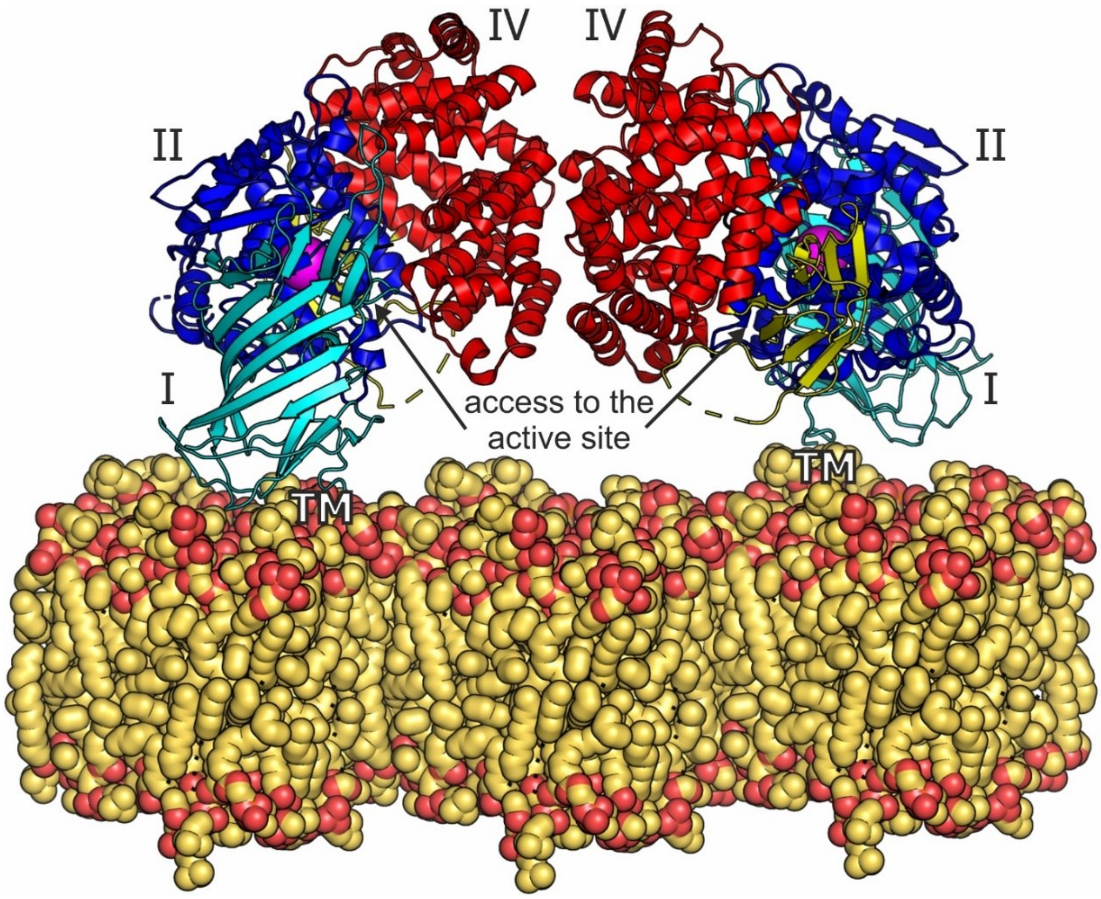

Figure S4. Conceptual model of an IRAP dimer on the cell membrane. The dimer is constructed based on the X-ray structure of the extracellular part of IRAP, oriented so that the N-terminal transmembrane (TM) helix is inserted into a lipid bilayer model. The hinge domain III is colored yellow and domains I, II and IV are labelled. The open state of IRAP allows access of substrates to the active site as shown by the two arrows (the catalytic Zn(II) atom is indicated with a magenta sphere).

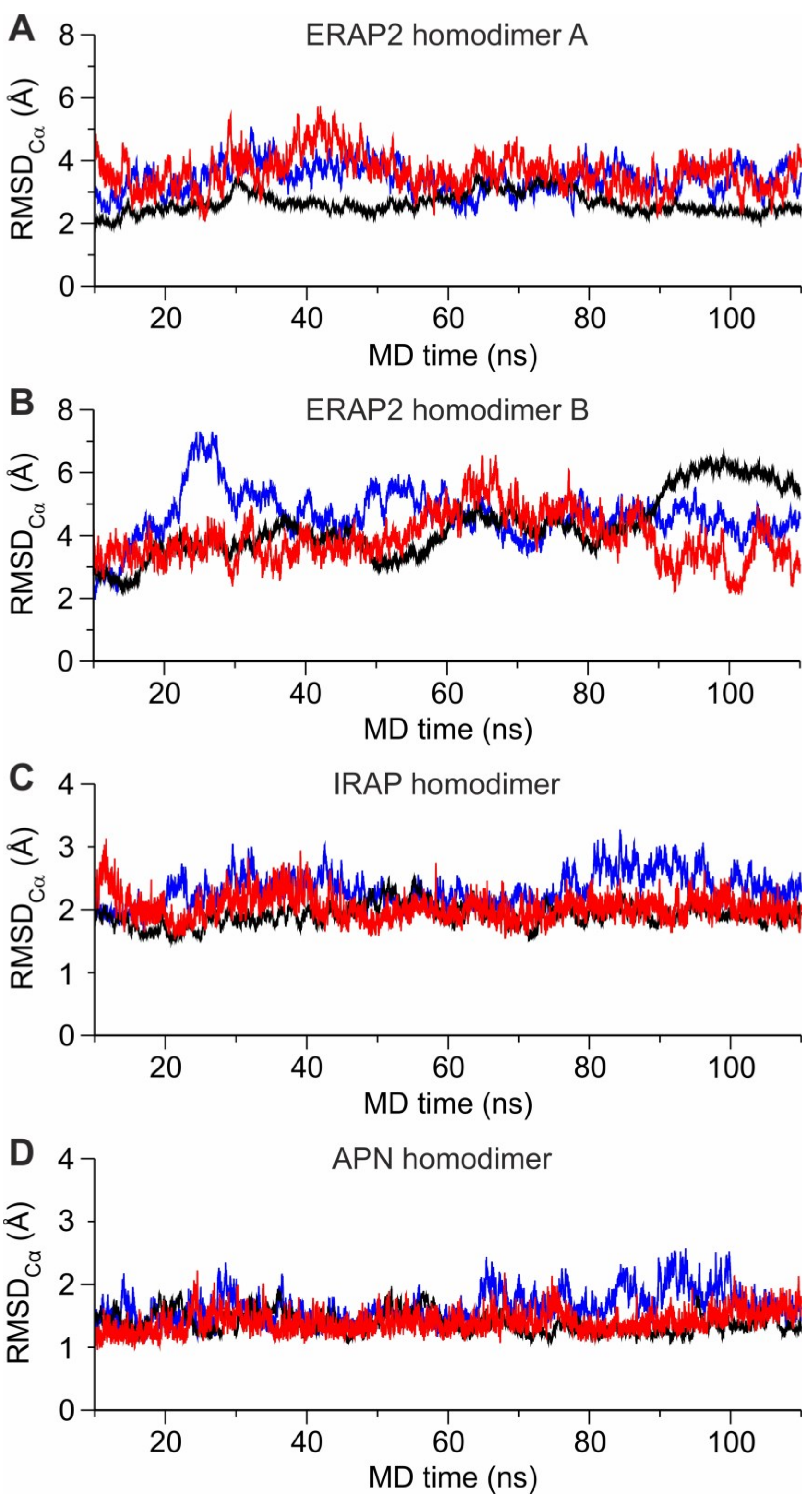

Figure S5. Plots of the root-mean-square deviation (RMSD) of all protein C^{α} atoms from the initial model as a function of MD simulation time. The plots show only the triplicate production runs between 10–110 ns, excluding the initial 10 ns of equilibration. The two ERAP2 homodimeric topologies are designated as described in the main text (**Table 1**, **Figure 1**).

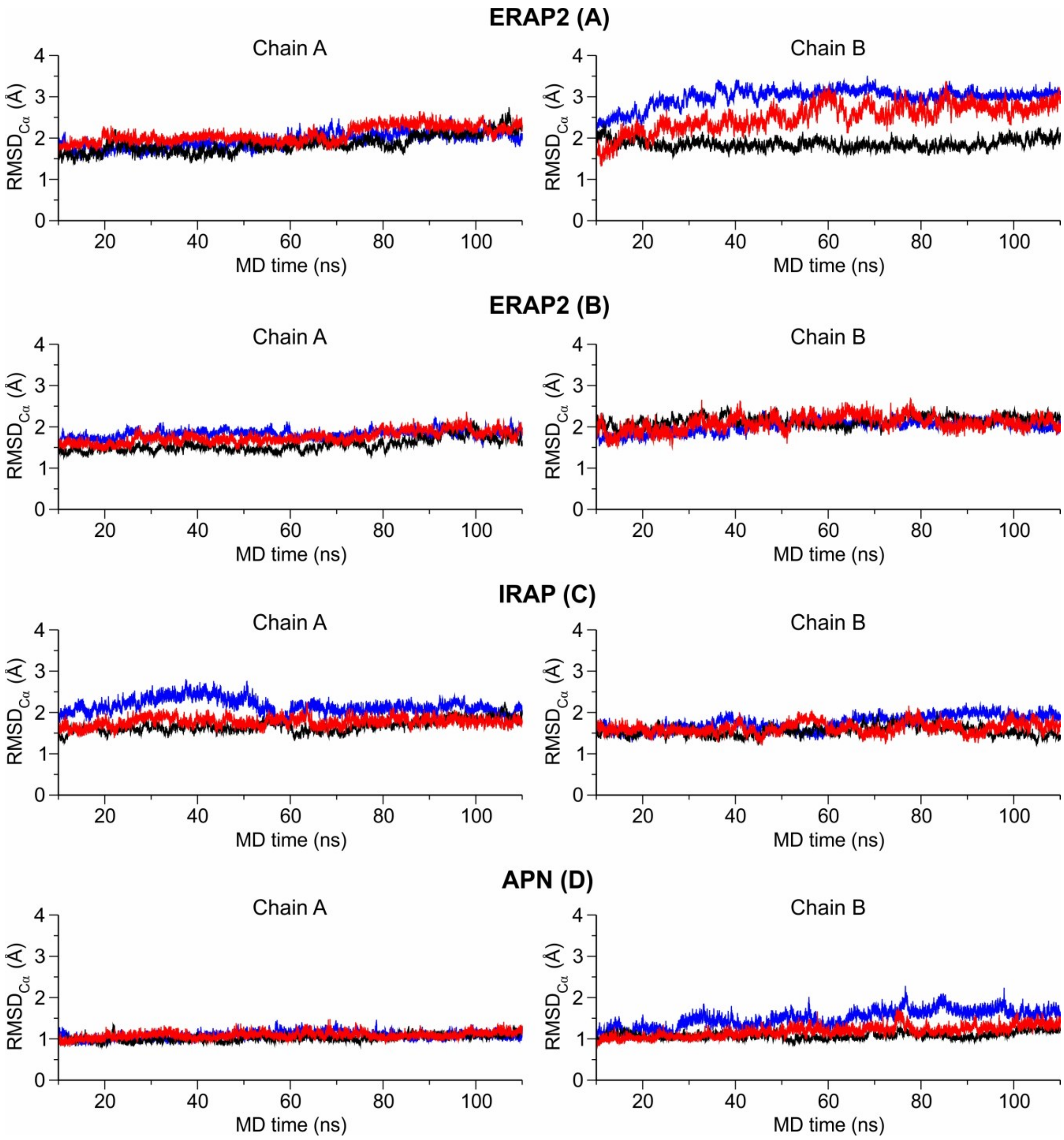

Figure S6. Plots of the root-mean-square deviation (RMSD) from the initial model using all protein C^{α} atoms of each protomer as a function of MD simulation time. The plots show only the triplicate production runs between 10–110 ns, excluding the initial 10 ns of equilibration.

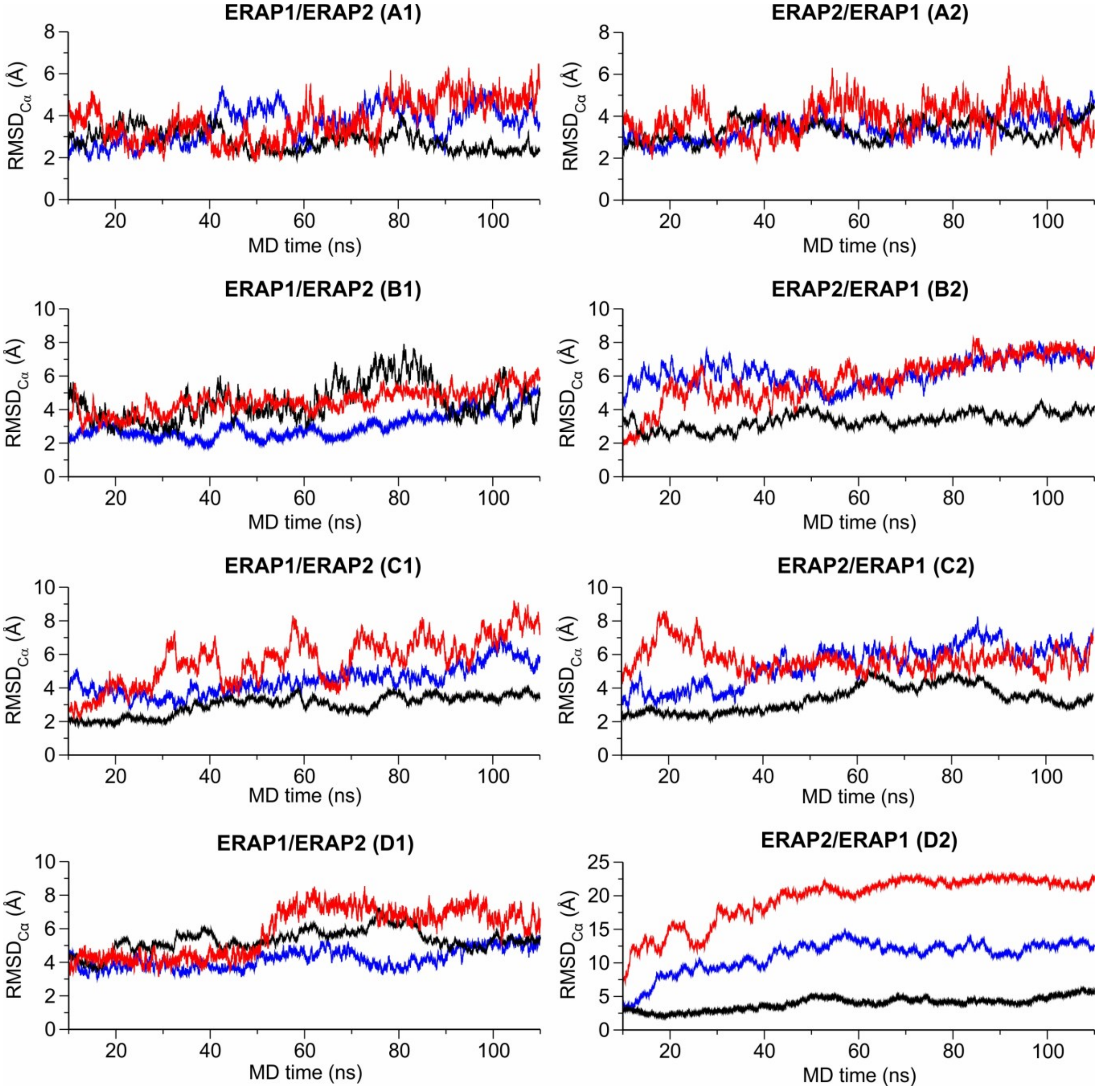

Figure S7. Plots of the root-mean-square deviation (RMSD) of all protein C^{α} atoms from the initial model as a function of MD simulation time. The plots show only the triplicate production runs between 10–110 ns, excluding the initial 10 ns of equilibration. Each heterodimeric system is designated as shown in the main text **Table 1** and **Figures 1, 2**.

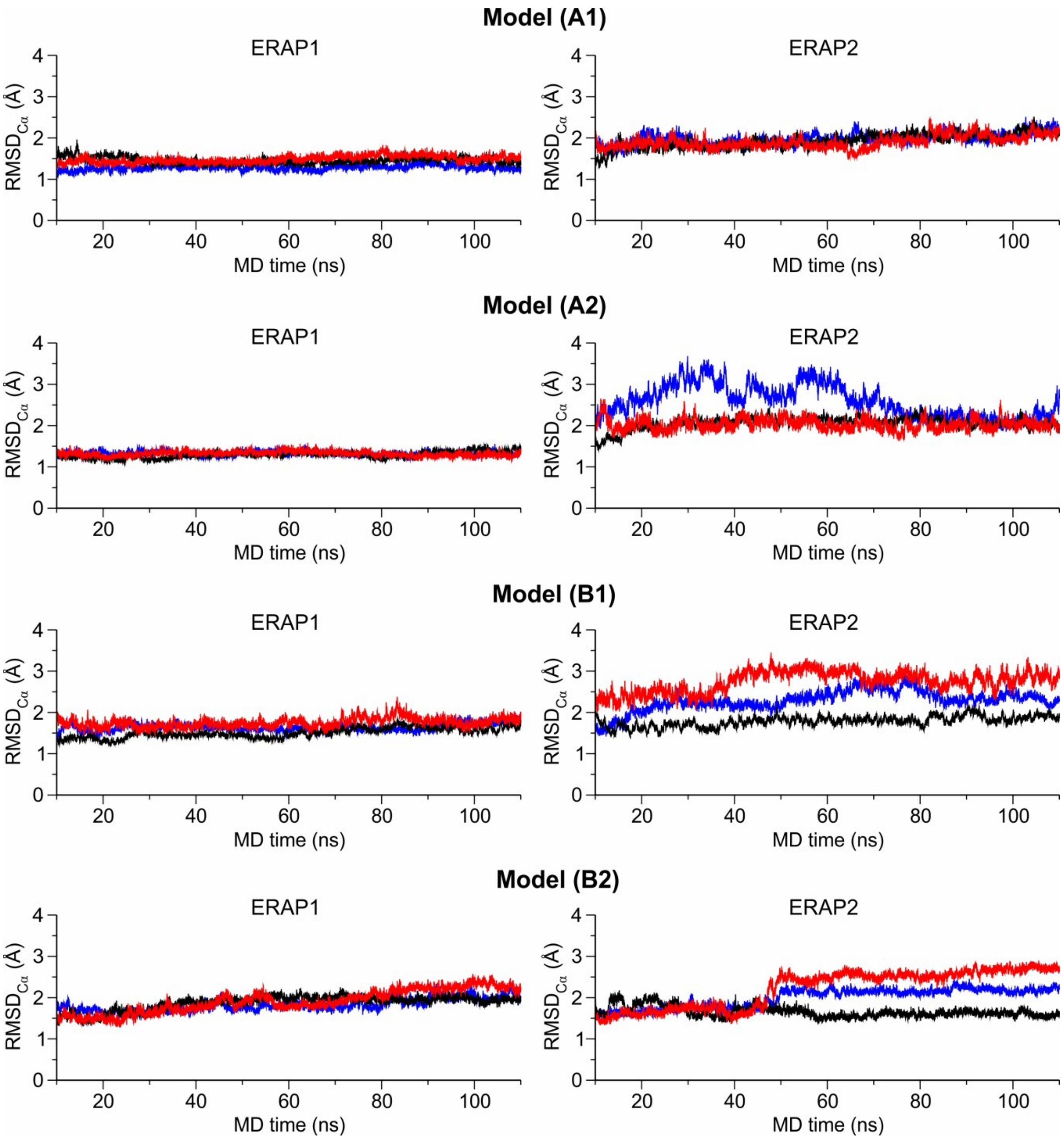

Figure S8. Plots of the root-mean-square deviation (RMSD) from the initial model using protein C^{α} atoms of each protomer as a function of MD simulation time. The plots show only the triplicate production runs between 10–110 ns, excluding the initial 10 ns of equilibration.

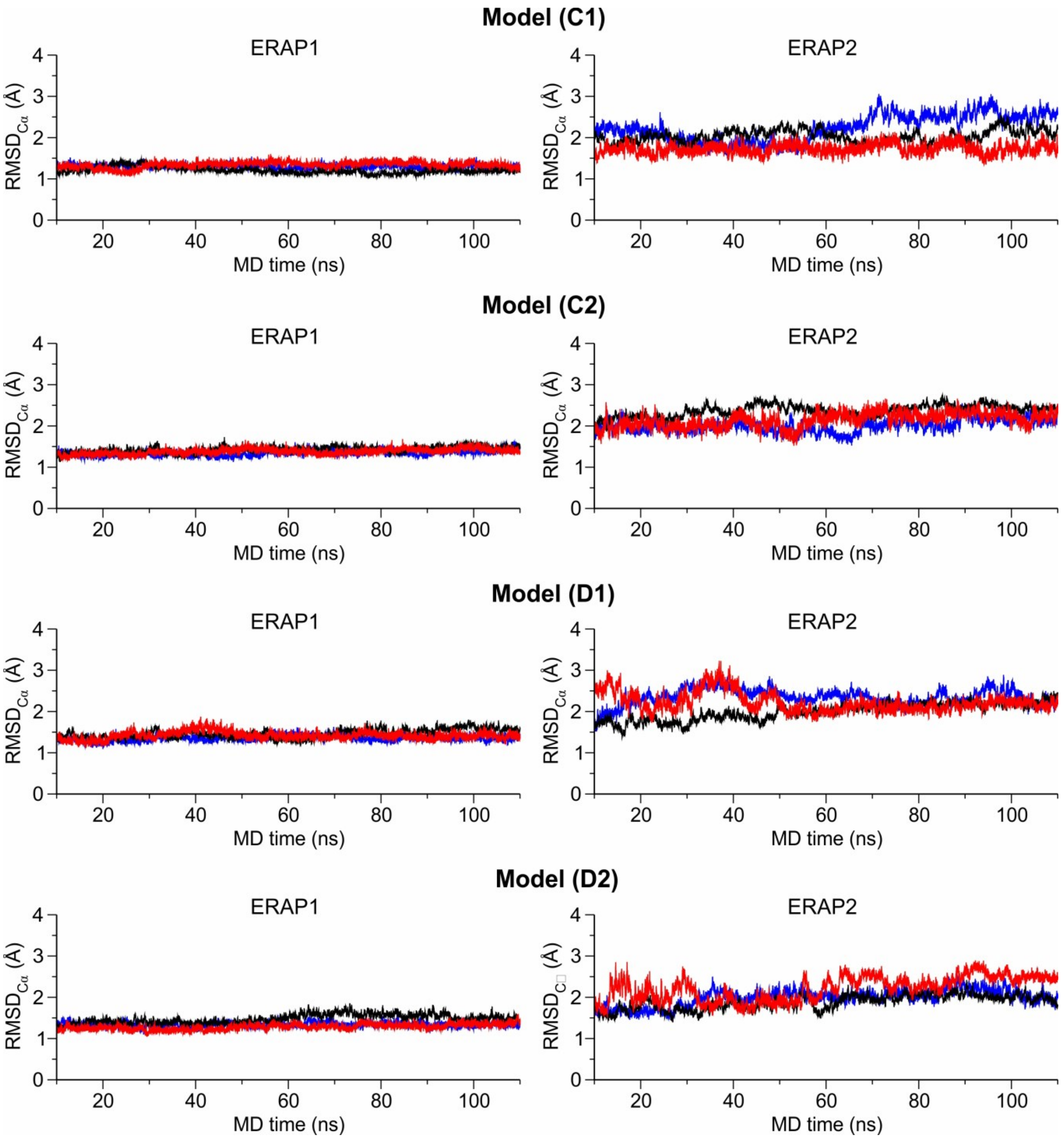

Figure S8 (cont.) Plots of the root-mean-square deviation (RMSD) from the initial model using protein C^{α} atoms of each protomer as a function of MD simulation time. The plots show only the triplicate production runs between 10–110 ns, excluding the initial 10 ns of equilibration.

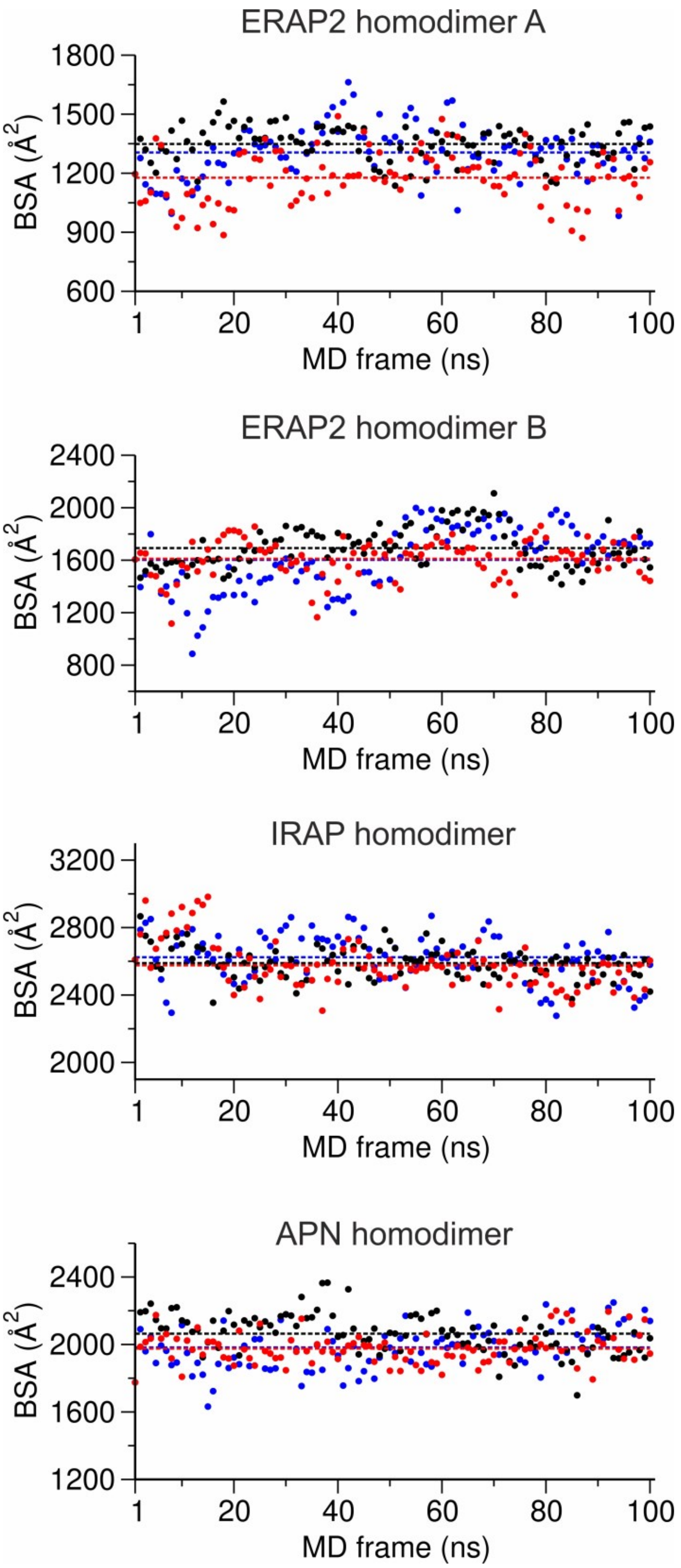

Figure S9. Plots of the buried surface area (BSA) as a function of MD frame that was equally sampled every 1.0 ns from the 100-ns production MD runs performed in triplicate. Each system is designated, whereas the dashed lines indicate the mean value of BSA for each simulation.

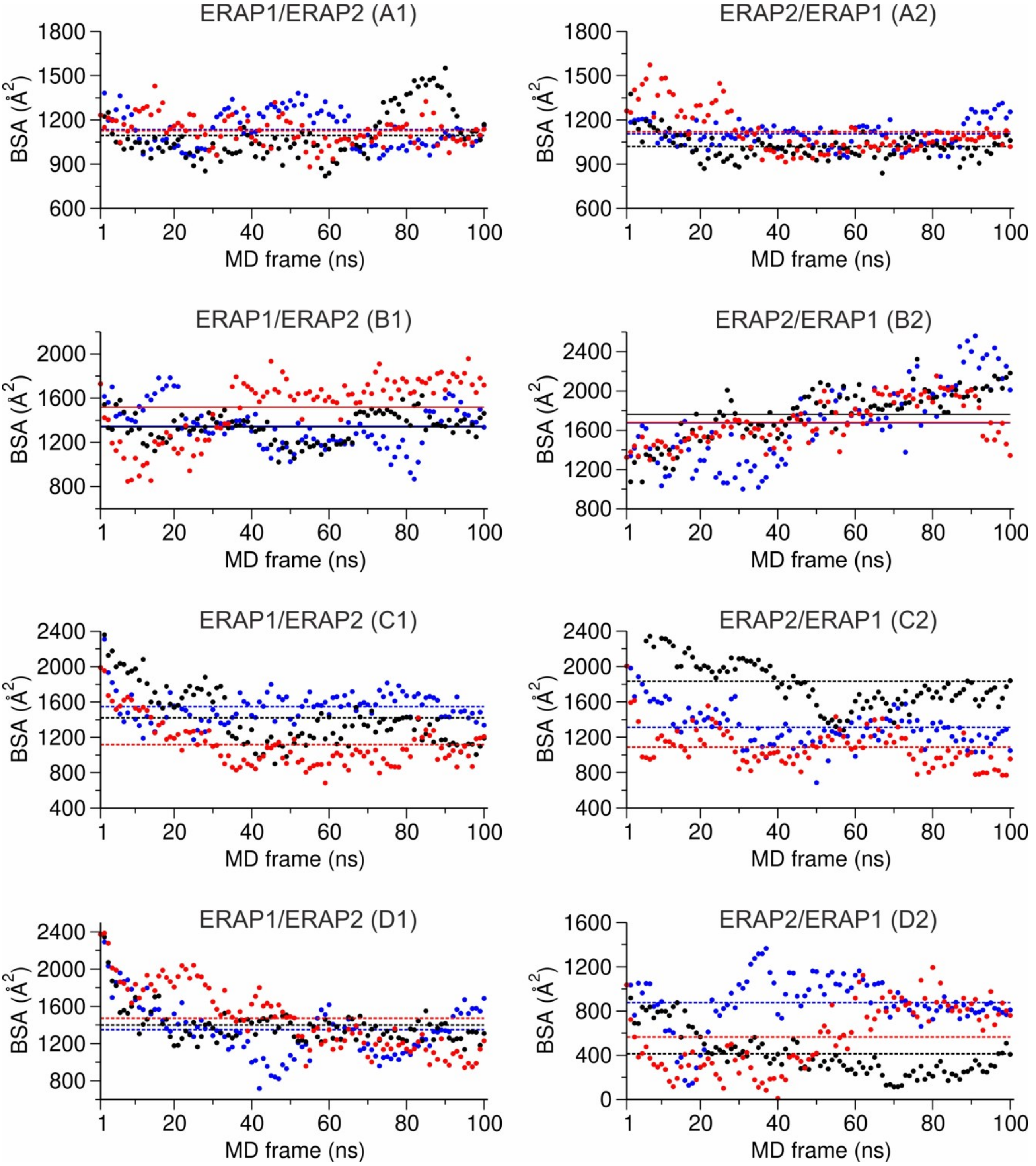

Figure S10. Plots of the buried surface area (BSA) as a function of MD frame sampled every 1.0 ns from 100-ns production MD runs that were performed in triplicate. Each heterodimeric system is designated as in the main text (**Table** 1), whereas the dashed lines indicate the mean value of the BSA for each simulation.

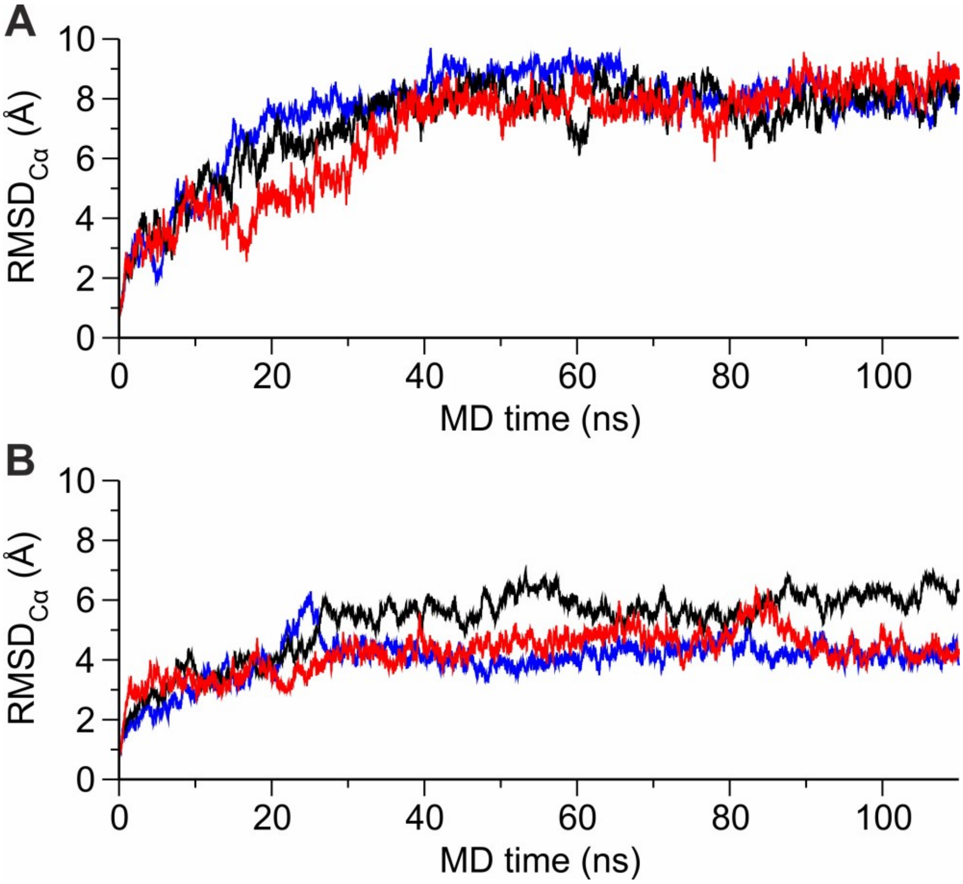

Figure S11. A) Plots of the root-mean-square deviation (RMSD) for the C^{α} atoms of the ERAP1/ERAP2 dimer from the initial structure as a function of simulation time. Three simulations have been performed for ERAP1–Jun/ERAP2–Fos dimer that was prepared based on the heterodimeric topology of model B2. B) The same plot as in (A) from three simulations of ERAP1–Jun/ERAP2–Fos dimer that was prepared based on the heterodimeric topology of model C2.

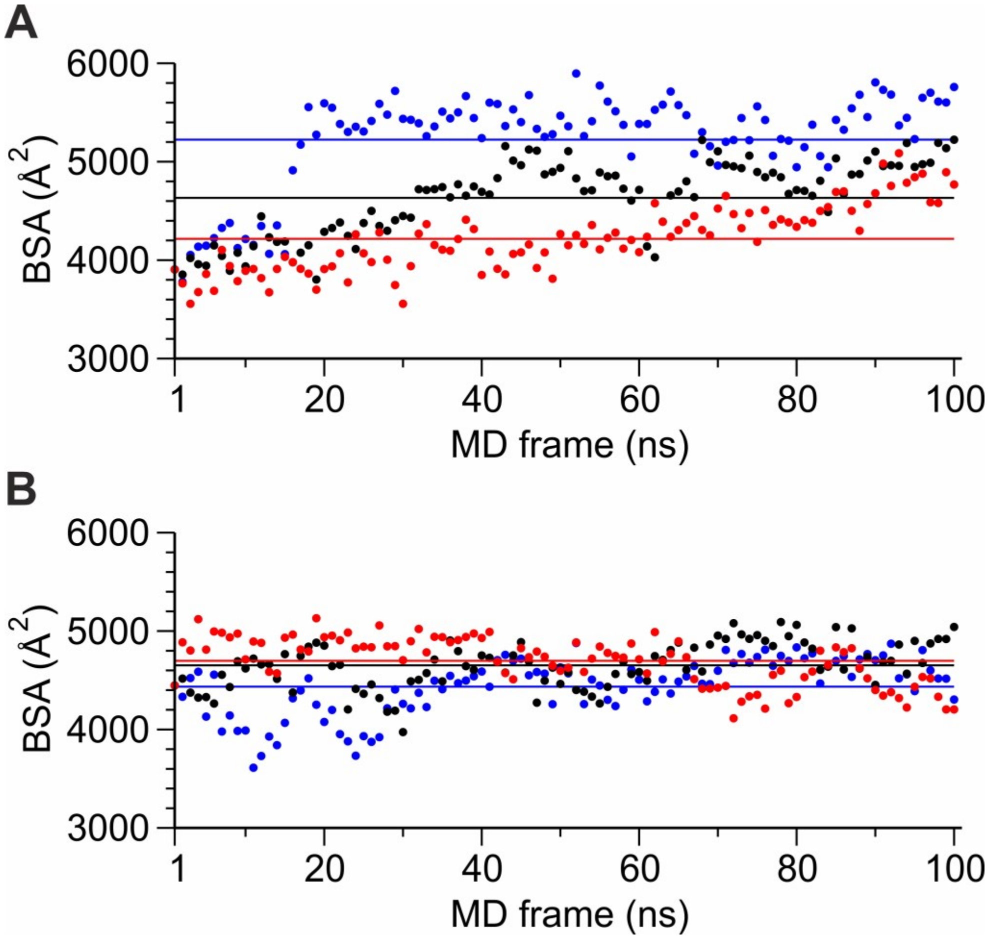

Figure S12. A) Plots of the total buried surface area (BSA) of ERAP1–Jun/ERAP2–Fos dimer that was based on the heterodimeric topology of model B2. Three simulations have been performed and shown in different color with the horizontal line indicating the mean value. B) The same plots as in (A) from three simulations of ERAP1–Jun/ERAP2–Fos dimer that was based on the heterodimeric topology of model C2.

Table S2. Contacts between ERAP1 and ERAP2 as observed in representative snapshots of the most populated cluster that were taken from characteristic MD simulations of the heterodimeric models. Contacts were calculated using the DIMPLOT module of LIGPLOT [1], with cutoffs at 3.5 Å and 4.0 Å between heavy atoms for hydrogen bonding and non-bonded contacts, respectively. Ionic interactions are highlighted.

Model B2

Hydrogen bonds

ERAP1			ERAP2			Å
GLU	476	OE2	LYS	519	NZ	2.69
GLU	476	OE1	MET	520	N	2.78
GLN	512	OE1	HIS+	515	NE2	2.63
ASP	516	OD2	LYS	484	NZ	2.75
ASN	522	OD1	THR	521	N	3.07
ASN	522	ND2	THR	521	O	2.83
ARG	540	NH1	GLU	66	OE1	3.38
ARG	540	NH2	GLU	66	OE1	3.46
ASP	575	OD2	ASN	103	ND2	3.01

Non-bonded contacts

ERAP1			ERAP2			Å
ASN	475	ND2	MET	520	CG	3.57
GLU	476	OE1	LYS	519	CA	3.30
GLU	476	OE1	LYS	519	CB	3.96
GLU	476	OE1	LYS	519	CD	3.92
GLU	476	OE1	LYS	519	CE	3.33
GLU	476	OE2	LYS	519	CE	3.76
GLU	476	CD	LYS	519	NZ	3.26
GLU	476	OE1	LYS	519	NZ	3.01
GLU	476	OE1	LYS	519	C	3.47
GLU	476	CD	MET	520	N	3.67
GLU	476	OE1	MET	520	CA	3.68
GLU	476	CG	MET	520	CB	3.39
GLU	476	CD	MET	520	CB	3.81
GLU	476	OE1	MET	520	CB	3.43
TRP	479	CD1	MET	520	CE	3.26
TRP	479	NE1	MET	520	CE	3.58
SER	508	OG	ILE	316	CD1	3.79
SER	508	CB	LYS	483	CB	3.92
SER	508	O	LYS	483	CB	3.52
SER	508	O	LYS	483	C	3.71
SER	508	CB	LYS	483	O	3.94
SER	508	C	LYS	483	O	3.53
SER	508	O	LYS	483	O	3.54
HIS	509	N	LYS	483	O	3.38
HIS	509	CA	LYS	483	O	3.50
HIS	509	CB	LYS	483	O	3.44
HIS	509	CB	LYS	484	C	3.99

Non-bonded contacts

ERAP1			ERAP2			Å
HIS	509	CB	LYS	484	O	3.16
HIS	509	CB	SER	486	OG	3.81
HIS	509	ND1	SER	486	OG	3.78
HIS	509	NE2	ARG	488	CZ	3.74
HIS	509	NE2	ARG	488	NH1	3.32
HIS	509	CD2	ARG	488	NH1	3.23
HIS	509	CE1	ARG	488	NH2	3.99
HIS	509	NE2	ARG	488	NH2	3.56
HIS	511	CE1	SER	516	CB	3.81
HIS	511	NE2	SER	516	CB	3.51
HIS	511	NE2	SER	516	OG	3.34
GLN	512	CD	GLN	480	CG	3.74
GLN	512	OE1	GLN	480	CG	3.68
GLN	512	NE2	GLN	480	CG	3.88
GLN	512	OE1	HIS+	515	CE1	3.59
GLN	512	CD	HIS+	515	NE2	3.80
GLN	512	OE1	HIS+	515	CD2	3.61
VAL	515	CG1	HIS+	515	CE1	3.60
VAL	515	CG1	HIS+	515	O	3.63
VAL	515	CG2	HIS+	515	O	3.85
ASP	516	OD2	LYS	484	CE	3.78
ASP	516	CG	LYS	484	NZ	3.82
THR	519	CA	MET	524	SD	3.66
THR	519	CG2	MET	524	SD	3.77
THR	519	O	MET	524	SD	3.89
THR	519	CG2	MET	524	CE	3.44
THR	519	O	MET	524	CE	3.61
ASN	522	OD1	MET	520	CA	3.44
ASN	522	N	MET	520	SD	3.86
ASN	522	CA	MET	520	SD	3.50
ASN	522	CB	MET	520	SD	3.87
ASN	522	CG	MET	520	SD	3.53
ASN	522	OD1	MET	520	SD	3.58
ASN	522	ND2	MET	520	SD	3.94
ASN	522	N	MET	520	CE	3.53
ASN	522	CA	MET	520	CE	3.87
ASN	522	OD1	MET	520	C	3.66
ASN	522	CG	THR	521	N	3.72
ASN	522	ND2	THR	521	N	3.59

Non-bonded contacts

ERAP1			ERAP2			Å
ASN	522	ND2	THR	521	C	3.61
ASN	522	CG	THR	521	O	3.70
ASN	522	OD1	THR	521	O	3.67
ASN	522	CG	MET	524	CB	3.90
ASN	522	OD1	MET	524	CB	3.37
ASN	522	OD1	MET	524	CG	3.71
ASN	522	CB	MET	524	SD	3.82
ASN	522	CG	MET	524	SD	3.69
ASN	522	OD1	MET	524	SD	3.31
ASN	522	ND2	LEU	525	CG	3.53
ASN	522	O	LEU	525	CG	3.94
ASN	522	ND2	LEU	525	CD1	3.67
ASN	522	O	LEU	525	CD1	3.37
ASN	522	ND2	LEU	525	CD2	3.87
ASN	522	C	LEU	525	CD2	3.83
ASN	522	O	LEU	525	CD2	3.61
THR	523	N	LEU	525	CD2	3.92

Non-bonded contacts

ERAP1			ERAP2			Å
THR	523	CA	LEU	525	CD2	3.76
THR	523	CG2	LEU	525	CD2	3.56
THR	523	CG2	LEU	528	CD2	3.97
LEU	526	CD1	THR	521	N	3.84
LEU	526	CD1	THR	521	CA	3.72
LEU	526	CD1	THR	521	C	3.65
LEU	526	CD1	SER	522	N	3.83
LEU	526	CB	LEU	525	CD1	3.94
LEU	526	O	LEU	525	CD1	3.95
GLN	527	CG	LEU	525	CD1	3.90
ARG	540	CZ	GLU	66	OE1	3.88
HIS	564	CE1	LEU	528	CD1	3.90
HIS	564	NE2	LEU	528	CD1	3.12
HIS	564	NE2	LEU	528	CD2	3.64
ASP	575	OD2	ASN	103	CB	3.76
ASP	575	OD2	ASN	103	CG	3.86
MET	576	SD	HIS	159	CE1	3.56

Model A2

Hydrogen bonds

ERAP1			ERAP2			Å
GLU	171	OE1	ARG	229	NH1	2.82
GLU	171	OE2	ARG	229	NH2	2.76
GLU	173	OE2	ARG	229	NH1	3.07
LEU	174	O	THR	191	N	2.87
LEU	174	N	THR	191	O	3.23
ILE	176	N	GLU	190	OE1	2.78
ARG	212	NH1	GLU	190	OE2	2.73
ARG	212	NH2	GLU	190	OE2	3.12

Non-bonded contacts

ERAP1			ERAP2			Å
THR	72	O	GLY	188	N	3.84
THR	72	CA	GLY	188	CA	3.92
THR	72	O	GLY	188	CA	3.37
GLU	171	OE2	LEU	86	CB	3.47
GLU	171	CG	LEU	86	C	3.68
GLU	171	OE2	LEU	86	C	3.92
GLU	171	CG	LEU	86	O	3.64
GLU	171	CG	THR	87	N	3.88
GLU	171	OE2	THR	87	N	3.86
GLU	171	CG	THR	87	CA	3.92
GLU	171	CG	THR	87	OG1	3.86
GLU	171	CD	THR	87	OG1	3.74
GLU	171	OE2	THR	87	OG1	3.43

Non-bonded contacts

ERAP1			ERAP2			Å
GLU	171	O	LYS	181	NZ	3.75
GLU	171	O	ILE	193	CD1	3.69
GLU	171	CD	ARG	229	CZ	3.98
GLU	171	OE1	ARG	229	CZ	3.29
GLU	171	OE2	ARG	229	CZ	3.85
GLU	171	CD	ARG	229	NH1	3.85
GLU	171	CD	ARG	229	NH2	3.14
GLU	171	OE1	ARG	229	NH2	2.84
GLY	172	O	THR	191	CG2	3.63
GLY	172	C	ILE	193	CD1	3.80
GLU	173	CA	THR	191	O	3.52
GLU	173	CB	THR	191	O	3.57
GLU	173	CG	THR	191	O	3.83
GLU	173	C	THR	191	O	3.80
GLU	173	N	ILE	193	CD1	3.82
GLU	173	CD	ARG	229	NH1	3.48
GLU	173	OE1	ARG	229	NH1	3.45
LEU	174	O	GLU	190	CA	2.96
LEU	174	O	GLU	190	CB	3.13
LEU	174	O	GLU	190	CG	3.26
LEU	174	O	GLU	190	C	3.37
LEU	174	N	THR	191	N	3.80
LEU	174	CA	THR	191	N	3.88

Non-bonded contacts

ERAP1			ERAP2			Å
LEU	174	CB	THR	191	N	3.66
LEU	174	C	THR	191	N	3.79
LEU	174	N	THR	191	CA	3.89
LEU	174	O	THR	191	CA	3.98
LEU	174	N	THR	191	CB	3.55
LEU	174	CB	THR	191	CB	3.69
LEU	174	CB	THR	191	OG1	3.73
LEU	174	N	THR	191	C	3.99
LEU	174	O	THR	191	O	3.79
ARG	175	CA	GLU	190	OE1	3.50
ARG	175	CB	GLU	190	OE1	3.97
ARG	175	C	GLU	190	OE1	3.66
ARG	175	NH2	ARG	229	CZ	3.99
ARG	175	NH2	ARG	229	NH1	3.20

Non-bonded contacts

ERAP1			ERAP2			Å
ILE	176	CG1	GLY	188	O	3.77
ILE	176	N	GLU	190	CG	3.78
ILE	176	CG1	GLU	190	CG	3.84
ILE	176	N	GLU	190	CD	3.67
ILE	176	CA	GLU	190	OE1	3.66
ILE	176	CB	GLU	190	OE1	3.76
ILE	176	O	GLU	190	OE1	3.55
ARG	212	NH1	GLU	190	CD	3.39
ARG	212	NH1	GLU	190	OE1	3.27
ARG	212	CZ	GLU	190	OE2	3.31
ARG	212	NE	ARG	192	NH2	3.94
ARG	212	CZ	ARG	192	NH2	3.24
ARG	212	NH1	ARG	192	NH2	3.34
ARG	212	NH2	ARG	192	NH2	3.23

Model C2

Hydrogen bonds

ERAP1			ERAP2			Å
ASP	691	OD1	ASN	879	ND2	2.89
ASN	693	OD1	LYS	839	NZ	2.73
THR	779	OG1	LYS	832	NZ	3.02
GLN	809	O	GLN	834	N	3.10
GLN	809	NE2	GLU	835	OE1	2.99
LYS	811	NZ	SER	831	O	2.58
LYS	811	N	LYS	832	O	3.13
GLU	812	OE1	LYS	867	NZ	2.84
GLU	812	OE2	LYS	867	NZ	2.67
ARG	854	NH1	GLU	913	OE1	2.83
THR	886	N	GLU	878	OE2	2.93
THR	886	OG1	GLU	878	OE2	2.76
ARG	887	NH2	ASP	874	OD1	2.89
GLU	890	OE1	ARG	877	NH1	2.76
GLU	890	OE1	ARG	877	NH2	2.83

Non-bonded contacts

ERAP1			ERAP2			Å
ASP	691	OD1	ASN	879	CG	3.60
ASP	691	CB	ASN	879	OD1	3.74
ASP	691	CG	ASN	879	OD1	3.75
ASP	691	OD1	ASN	879	OD1	3.35
ASP	691	CG	ASN	879	ND2	3.79
ASN	693	CG	LYS	839	CD	3.79
ASN	693	OD1	LYS	839	CD	3.41
ASN	693	ND2	LYS	839	CD	3.53
ASN	693	OD1	LYS	839	CE	3.57
ASN	693	CG	LYS	839	NZ	3.73

Non-bonded contacts

ERAP1			ERAP2			Å
ASN	693	CB	GLU	842	CG	3.88
ASN	693	OD1	GLU	842	CG	3.86
THR	779	OG1	LYS	832	CD	3.46
THR	779	OG1	LYS	832	CE	3.73
THR	779	CB	LYS	832	NZ	3.74
THR	779	CG2	LYS	832	NZ	3.53
THR	808	OG1	HIS	833	NE2	3.36
THR	808	OG1	HIS	833	CD2	3.25
GLN	809	C	HIS	833	CA	3.85
GLN	809	O	HIS	833	CA	3.35
GLN	809	O	HIS	833	CB	3.88
GLN	809	C	HIS	833	CD2	3.81
GLN	809	O	HIS	833	C	3.77
GLN	809	O	GLN	834	CB	3.86
GLN	809	CB	GLU	835	CD	3.84
GLN	809	NE2	GLU	835	CD	3.89
GLN	809	CB	GLU	835	OE1	3.46
ASN	810	ND2	LYS	832	CG	3.67
ASN	810	CA	LYS	832	O	3.35
ASN	810	CG	LYS	832	O	3.79
ASN	810	OD1	LYS	832	O	3.42
ASN	810	C	LYS	832	O	3.70
ASN	810	N	HIS	833	CD2	3.32
ASN	810	CA	HIS	833	CD2	3.63
ASN	810	CB	HIS	833	CD2	3.67
LYS	811	NZ	GLN	800	OE1	3.42
LYS	811	NZ	SER	831	C	3.62

Non-bonded contacts

ERAP1			ERAP2			Å
LYS	811	CD	SER	831	O	3.81
LYS	811	CE	SER	831	O	3.46
LYS	811	NZ	LYS	832	CA	3.59
LYS	811	CD	LYS	832	O	3.14
LYS	811	CB	GLN	834	CD	3.68
LYS	811	CB	GLN	834	OE1	3.81
LYS	811	CG	GLN	834	OE1	3.83
LYS	811	CB	GLN	834	NE2	3.62
LYS	811	O	LYS	867	CD	3.68
LYS	811	O	LYS	867	CE	3.39
GLU	812	OE1	LYS	867	CE	3.74
GLU	812	OE2	LYS	867	CE	3.64
GLU	812	CD	LYS	867	NZ	3.14
GLN	815	OE1	LYS	867	CE	3.25
GLN	815	OE1	LYS	867	NZ	3.62
VAL	844	CG1	GLU	835	N	3.71
VAL	844	CG1	GLU	835	CA	3.82
LEU	848	CD2	LYS	867	CD	3.90
LEU	848	CD2	LYS	867	CE	3.88
ARG	854	NH2	GLN	870	OE1	3.68
ARG	854	NH2	LYS	910	NZ	3.31
ARG	854	CZ	GLU	913	OE1	3.76
THR	884	CB	GLU	878	CD	3.59
THR	884	CB	GLU	878	OE1	3.46
ARG	885	N	GLU	878	OE1	3.84
ARG	885	N	GLU	878	OE2	3.67

Non-bonded contacts

ERAP1			ERAP2			Å
THR	886	CG2	ARG	877	CB	3.86
THR	886	CG2	ARG	877	NE	3.68
THR	886	CG2	ARG	877	CZ	3.54
THR	886	CG2	ARG	877	NH1	3.47
THR	886	OG1	ARG	877	C	3.88
THR	886	OG1	GLU	878	N	3.53
THR	886	OG1	GLU	878	CA	3.37
THR	886	OG1	GLU	878	CB	3.62
THR	886	OG1	GLU	878	CD	3.83
THR	886	CA	GLU	878	OE2	3.66
THR	886	CB	GLU	878	OE2	3.23
ARG	887	NH2	ASP	874	CB	3.34
ARG	887	CZ	ASP	874	CG	3.75
ARG	887	NH1	ASP	874	CG	3.54
ARG	887	NH2	ASP	874	CG	3.18
ARG	887	CZ	ASP	874	OD1	3.63
ARG	887	NH1	ASP	874	OD1	3.40
ARG	887	NH1	ASP	874	OD2	3.47
ARG	887	NH2	ASP	874	OD2	3.82
GLU	890	OE1	ARG	877	CZ	3.27
GLU	890	CD	ARG	877	NH1	3.84
GLU	890	OE1	GLU	913	CD	3.56
GLU	890	CG	GLU	913	OE1	3.89
GLU	890	CD	GLU	913	OE1	3.85
GLU	890	OE1	GLU	913	OE1	3.16
GLU	890	OE1	GLU	913	OE2	3.82

Model D1

Hydrogen bonds

ERAP1			ERAP2			Å
ASP	691	OD2	LYS	867	NZ	2.74
ASN	693	N	GLU	835	OE2	3.16
ASN	693	ND2	GLN	834	O	2.98
GLN	851	OE1	LYS	910	NZ	2.78
GLU	890	OE2	ARG	877	NH1	2.87

Non-bonded contacts

ERAP1			ERAP2			Å
ASP	691	CG	GLN	834	OE1	3.83
ASP	691	CG	LYS	867	NZ	3.14
ASP	691	OD1	LYS	867	NZ	2.95
ASP	691	OD1	GLN	870	CD	3.95
ASP	691	OD1	GLN	870	OE1	3.67
ASP	691	OD2	GLN	834	OE1	3.79
ASP	691	OD2	LYS	867	CE	3.27

Non-bonded contacts

ERAP1			ERAP2			Å
ASP	691	O	GLU	835	CG	3.93
MET	692	CA	GLU	835	OE2	3.92
ASN	693	N	GLU	835	CG	3.87
ASN	693	N	GLU	835	CD	3.86
ASN	693	CB	GLN	834	O	3.43
ASN	693	CB	GLU	835	OE2	3.69
ASN	693	CG	GLN	834	O	3.62
ASN	693	ND2	LYS	836	CG	3.99
LYS	811	CE	ARG	713	O	3.39
LYS	811	C	ASN	714	O	3.75
LYS	811	O	ASN	714	O	3.53
GLU	812	N	ASN	714	O	3.77
GLU	812	CA	ASN	714	O	3.34
GLU	812	CG	ASN	714	C	3.98
GLU	812	CG	ASN	714	O	3.98

Non-bonded contacts

ERAP1			ERAP2			Å
GLU	812	CG	ILE	715	N	3.78
GLU	812	CG	ILE	715	CA	3.92
GLU	812	CG	ILE	715	C	3.77
GLU	812	CG	SER	716	N	3.77
GLU	812	CG	SER	716	O	3.34
GLU	812	CD	ASN	714	C	3.82
GLU	812	CD	ILE	715	N	3.91
GLU	812	OE2	ARG	713	C	3.98
GLU	812	OE2	ARG	713	O	3.40
GLU	812	OE2	ASN	714	N	3.95
GLU	812	OE2	ASN	714	CA	3.42
GLU	812	OE2	ASN	714	C	3.24
GLU	812	OE2	ASN	714	O	3.77
GLU	812	OE2	ILE	715	N	3.34
GLN	815	CB	ASN	714	O	3.53
GLN	815	CG	ASN	714	O	3.95

Non-bonded contacts

ERAP1			ERAP2			Å
GLN	815	CG	ILE	715	CB	3.94
LEU	848	CD2	ASN	714	CG	3.60
LEU	848	CD2	ASN	714	OD1	3.86
LEU	848	CD2	ASN	714	ND2	3.61
GLN	851	CD	LYS	910	NZ	3.75
GLN	851	OE1	LYS	910	CE	3.66
GLN	851	OE1	GLU	913	OE2	3.83
GLN	851	NE2	ASP	909	CG	3.95
GLN	851	NE2	ASP	909	OD2	3.41
THR	886	OG1	GLU	878	CD	3.92
THR	886	OG1	GLU	878	OE1	3.94
THR	886	OG1	GLU	878	OE2	3.80
GLU	890	CD	ARG	877	NH1	3.95
GLU	890	OE2	ARG	877	CZ	3.73
GLU	890	OE2	ARG	877	NH2	3.78

[1] Wallace A C, Laskowski R A & Thornton J M (1995). LIGPLOT: A program to generate schematic diagrams of protein-ligand interactions. Prot. Eng., 8, 127-134. DOI: 10.1093/protein/8.2.127
